# Arousal-driven critical roaming reproduces human functional connectivity dynamics

**DOI:** 10.1371/journal.pbio.3003916

**Published:** 2026-09-01

**Authors:** Anagh Pathak, Demian Battaglia

**Affiliations:** Laboratoire de Neurosciences Cognitives et Adaptatives (LNCA), Université de Strasbourg, CNRS, UMR 7364, Strasbourg, France; Sungkyunkwan University, REPUBLIC OF KOREA

## Abstract

Ongoing brain activity displays rich temporal variability associated with efficient cognition, with functional connectivity (FC) continually reconfiguring over time. The resulting functional connectivity dynamics (FCD) specifically show complex, fat-tailed statistics that alternate between persistent epochs and faster reconfiguration transients. While nonlinear whole-brain models tuned nearby a critical point have reproduced some aspects of FCD, they fall short of capturing its full temporal complexity. We propose that slow fluctuations in arousal offer a biologically plausible mechanism for exploring critical regimes in large-scale brain dynamics and thus enrich FCD. Using a connectome-based model of coupled cortical populations, we identified phase boundaries where system dynamics transition between regimes of faster or slower FCD. We then phenomenologically incorporated arousal changes, modeling them as stochastic fluctuations in key parameters such as cortical excitability, input gain, and noise amplitude. This explicitly time-dependent formulation enables the system to roam dynamically across regime boundaries, flexibly tuning its distance from critical transition lines and producing intermittent transitions that mirror the stochastic evolution observed in empirical FCD. Fitting these models to human resting-state fMRI and performing model comparison, we find that arousal-driven models more accurately reproduce the distinctive quantitative features of FCD, with the greatest improvements coming from the previously poorly accounted fat-tailed portions of the distributions. Together, these results suggest that arousal fluctuations—likely mediated by changes in neuromodulatory tone—shape the brain’s attractor landscape over time, expanding the repertoire of accessible functional network states and providing a mechanistic basis for the complexity of spontaneous functional dynamics.

## Abstract 2

What accounts for the highly dynamic patterns of brain activity at rest? This computational study shows that slow fluctuations in arousal help reproduce key features of brain regional communication dynamics, with implications for our understanding of critical brain states.

## Introduction

Even during quiet wakefulness, the mind is rarely still—its ongoing mentation drifts and alights like a bird in flight [1]. The neural counterpart of this restless stream of consciousness is echoed in Functional Connectivity (FC)—the pattern of correlated activity across distributed brain regions measured with fMRI—which itself fluctuates dynamically over time [2]. Much like the complex trajectories of real bird flight [3], the temporal evolution of FC can be conceptualized as a random walk through a high-dimensional connectivity space, where each step reflects the reconfiguration of large-scale network interactions [4–6]. Empirical analyses have shown that the distribution of FC step lengths deviates substantially from a Gaussian form, exhibiting fat-tailed statistics that signal complexity [4]. These dynamics alternate between “knots” and “leaps”, i.e., epochs of transient FC stabilization and rapid reconfiguration, respectively [4]. Importantly, such temporal complexity in functional connectivity dynamics (FCD) correlates with individual differences in aging and cognition [4,5,7,8] and can serve as a neuromarker of cognitive decline in neurodegenerative diseases [9–13].

To understand the origin of these statistical features, one must consider the underlying dynamical system that generates them. A helpful intermediate-level description is provided by the metaphor of an *attractor landscape*, in which brain activity evolves like a particle moving across a manifold whose geometry constrains its trajectory [14]. Within this framework, the fat-tailed distribution of FCD step lengths can arise naturally from multi- or metastable dynamics–such as heteroclinic cycles producing slow drifts punctuated by rapid transitions, or noise-enabled switching among stable FC configurations [15–18]. Such behaviors can emerge from the autonomous collective dynamics of coupled, stochastic, nonlinear neural populations, which constitute a dominant modeling approach to explain the temporal structure of FCD [15,17]. In this paradigm, structured FCD reflects operation at a single, fixed Dynamical Working Point (DWP), hypothesized to lie near a critical point of the system’s dynamics [19–21].

A complementary and less explored mechanism involves changes in *arousal*. Here, we use arousal to denote a global brain state that shapes vigilance and behavioral responsiveness through diffuse neuromodulatory influences on cortical activity and input integration. Multiple systems contribute to arousal [22–24], including cholinergic and noradrenergic pathways that project broadly across the cortex and can rapidly reshape the attractor landscape of neural dynamics [25]. Computational models linking neuromodulatory effects to biophysical parameters show that activation of these systems can shift the brain between more integrative or segregative network states [26]. Such transitions–potentially sharp even in response to smooth fluctuations in neuromodulatory tone–are facilitated by critical boundaries separating distinct dynamical regimes, with neuromodulatory inputs acting as control signals that steer the system’s DWP through parameter space. The primary control knobs of this process—parameters such as cortical excitability or neural gain—represent plausible sites of neuromodulatory action, enabling the brain to expand or contract its repertoire of accessible network configurations in a task-contingent manner, traversing critical transition lines with qualitative changes in dynamics.

In time-resolved FC studies, arousal is often treated as a nuisance factor; yet, unlike motion or physiological noise, arousal fluctuations have a genuine neural origin [27]. Recent animal work shows that arousal can be captured by a low-dimensional variable—such as pupil diameter—that explains a substantial fraction of ongoing, large-scale neural activity [28,29]. While large swings in arousal accompany sleep–wake transitions, more subtle modulations also occur during quiet wakefulness, suggesting that continuous arousal fluctuations may act as a latent driver of spontaneous network reorganization [30,31]. Extending the attractor metaphor, brain activity would no longer unfold on a static landscape; rather, the landscape itself would be continually reshaped as the system’s DWP drifts under the influence of fluctuating arousal, altering its instantaneous distance from the critical transition lines that structure the system’s collective behavior [32–35].

Here, we adopt a whole-brain computational modeling approach to assess whether dynamic complexity at a DWP near criticality is sufficient to generate structured and fluid FCD, or whether temporal fluctuations of the DWP are also required to account for the empirically observed organization of FCD. Specifically, we simultaneously fit multiple quantitative features of human resting-state fMRI FCD using two alternative families of connectome-based whole-brain models: first, autonomous dynamical systems in which activity unfolds on a fixed landscape; and second, nonautonomous (explicitly time-dependent), arousal-driven models in which the dynamical landscape itself changes over time through stochastic fluctuations in model parameters, mimicking arousal-related modulation.

We focus in particular on how well each model reproduces the fat tails of the empirical FCD fluidity and speed distributions, rather than just their mean values. Although both model classes capture general features of FCD, only the time-varying (nonautonomous) models naturally generate the very slow-speed events and the “viscous” reconfiguration patterns observed in empirical data—phenomena reflected in frustrated link-to-link interactions along FCD trajectories [11,12,36]. This is notable because precisely these reductions in dynamical fluidity—robust across multiple approaches yet poorly captured by previous modeling frameworks—have been proposed as markers of cognitive decline in aging, demanding task states, and neurodegenerative disease [4–6,11–13].

Our findings therefore suggest that intrinsic FCD is sculpted by ongoing fluctuations in neuromodulatory tone associated with arousal. They also refine earlier theories proposing that the brain sits near a single critical boundary. Instead, our fitted simulations indicate that the DWP roams across a broad inter-critical zone—spanning a large expanse along the ignition threshold [37]—giving rise to nonmonotonic changes in FCD fluidity as arousal varies.

## Results

**Fig 1:**
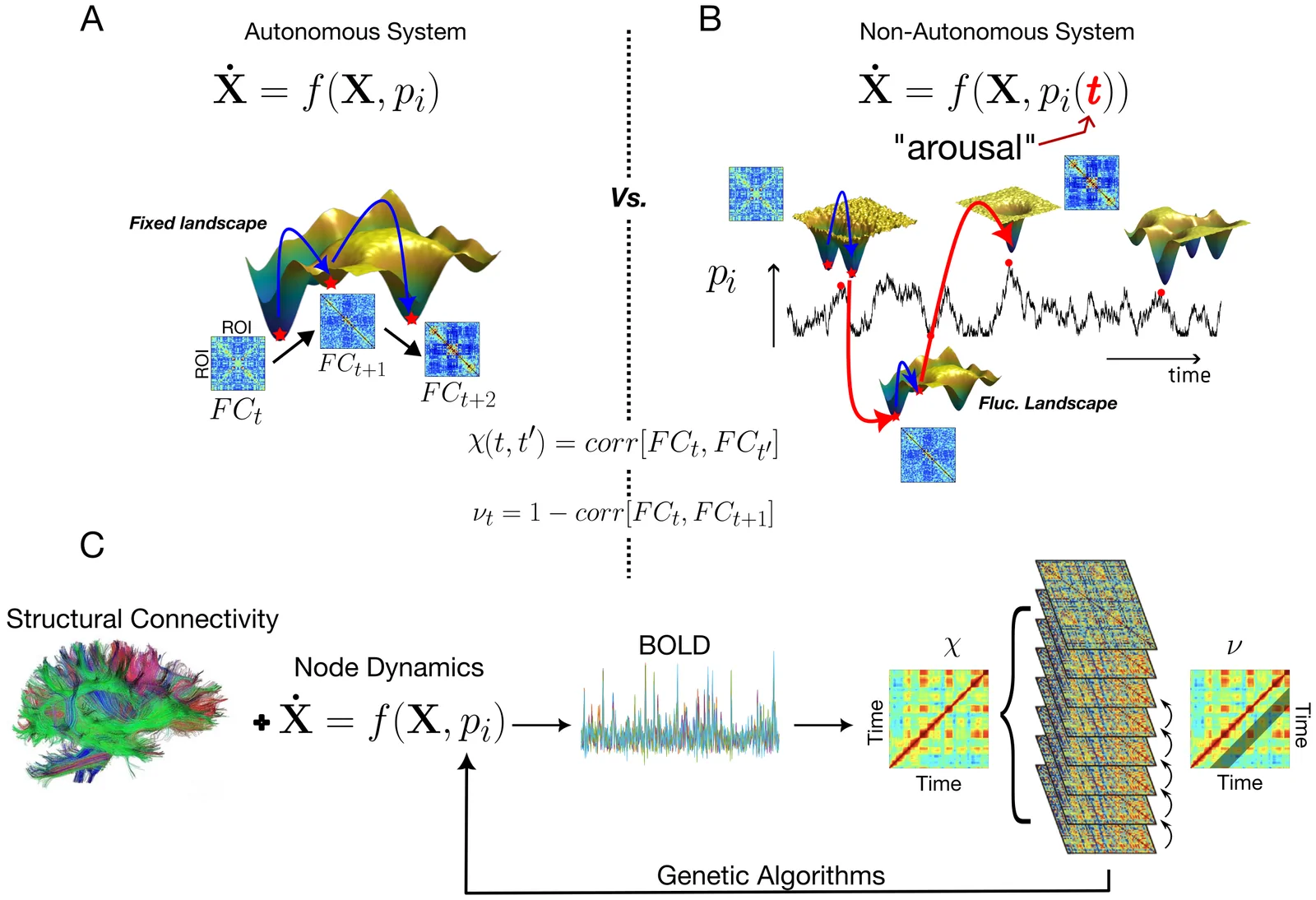
Calibrating autonomous vs. time-varying models to FCD statistics: we compare the performance of two classes of nonlinear models at capturing the temporal statistics of ongoing FCD as reflected in the distribution of FCD matrix entries (χ) and of sequential FCD variations, or FCD speed (ν). **A)** The activity of each brain region is described by autonomous ordinary differential equations (mean field model); each brain region receives inputs from other brain regions weighted by connection strength (as specified by diffusion imaging). The collective dynamics of this network give rise to FCD, whose variability is tracked by the entries (χ) of a recurrence matrix which gives the correlation between the windowed FCs across time frames. FCD speed (ν) represents the correlation distance between successive FC frames. The two metrics χ and ν together characterize FCD variability and its temporally ordered flow. In autonomous models, the working point of the system is fixed. **B)** To mimic the influence of arousal fluctuations, we use the same set of equations as in panel (A) but make some of the coefficients of the model time-dependent (fluctuating as an Ornstein-Uhlenbeck process). **C)** Using a Genetic Algorithm (Materials and Methods), the model was calibrated to a subset of resting-state fMRI recordings from the Human Connectome Project. The mean-field model’s internal parameters were optimized to align simulated dynamics with empirical observations, using the $5^{th},25^{th},50^{th},75^{th},95^{th}$ percentiles of χ and ν as fitting targets, to account simultaneously for typical as well as extreme transient behavior. For the nonautonomous (time-dependent) model, the parameters governing the Ornstein–Uhlenbeck process were additionally fitted.

Unlike previous approaches that segment FCD into discrete transitions between quasi-stable FC states [38], we describe FCD as a smooth, continuous flow through a space of continually morphing connectivity configurations [4]. Conventional analyses of static FC emphasize the spatial structure of connectivity networks while discarding most temporal information. An alternative perspective collapses each FC matrix into a single point in the high-dimensional space of all possible FC configurations—connectivity space. The erratic motion of this point through time can then be viewed as a stochastic trajectory or random walk across the manifold of possible network states. Most prior modeling studies have focused on reproducing the distribution of FCD values, while neglecting the sequential dependencies that define the temporal organization of FC reconfigurations [39,40]. Randomly shuffling the FCD streams, for instance, preserves the overall distribution but destroys its temporal structure—thus erasing the random-walk nature of the process [4]. To capture this essential aspect of FCD, we extend model fitting beyond the FCD distribution itself to include its step-length distribution, quantified as *FCD speed* (Fig 1). This metric characterizes how rapidly the system traverses connectivity space and the tails of this distribution encode rare but dynamically important transitions (corresponding to FCD “knots” and “leaps” [4]).

In the attempt to reproduce a rich FCD structure *in silico*, we first focus on a type of mean-field model (MFM) that has been extensively applied to characterize large-scale brain dynamics and reproduce key features of FC [15,40–42]. The model contains several parameters that can plausibly serve as control knobs for arousal, including cortical excitability and neural gain (which together set responsiveness and the relationship between synaptic input and population firing rate), the amplitude of stochastic noise, and the strength of local recurrent coupling within each neural population. Owing to a diversity of model parameters and nonlinear architecture, the MFM provides multiple avenues through which neuromodulatory input can transform system dynamics, making it a suitable platform for probing how arousal reshapes large-scale functional organization. In the following, we refer to the autonomous formulation as the MFM and to its time-dependent extension as tMFM, where *t* denotes the explicit temporal modulation introduced by arousal. Further, we label each tMFM variant according to the specific parameter endowed with temporal dependence: ${\text{tMFM}}_{G}$ for the global scaler of inter-regional coupling (*G*, coupling gain), ${\text{tMFM}}_{n}$ for background noise amplitude (σ), ${\text{tMFM}}_{a}$ for the gain parameter of regional sigmoidal response functions (*a*), and ${\text{tMFM}}_{w}$ for the intra-regional recurrent connectivity strength (*w*).

### Existence of critical transitions in FCD fluidity

Neuromodulatory systems are thought to dynamically tune the brain’s operating regime, enabling flexible transitions between distinct functional states [26,43]. The parameters of the MFM represent potential targets of such modulatory control. Given the nonlinear structure of the MFM, we further hypothesized that variations in key parameters could drive the system across critical boundaries, thereby reshaping the dynamic landscape of large-scale activity. To examine this, we asked how individual model parameters influence overall network dynamics and, in particular, the speed of fluctuations in FCD.

We systematically varied six model parameters—global coupling strength (*G*, also referred to as global excitability following [26]), noise amplitude (σ), local recurrent gain (*w*), and the three coefficients (*a*, *b*, *d*) governing the sigmoidal input–output function of each neural population (slope, threshold, and smoothness). Among these, *G* and σ represent global parameters controlling inter-regional coupling and stochastic drive, whereas *w*, *a*, *b*, and *d* define local circuit properties shared across regions [41].

**Fig 2:**
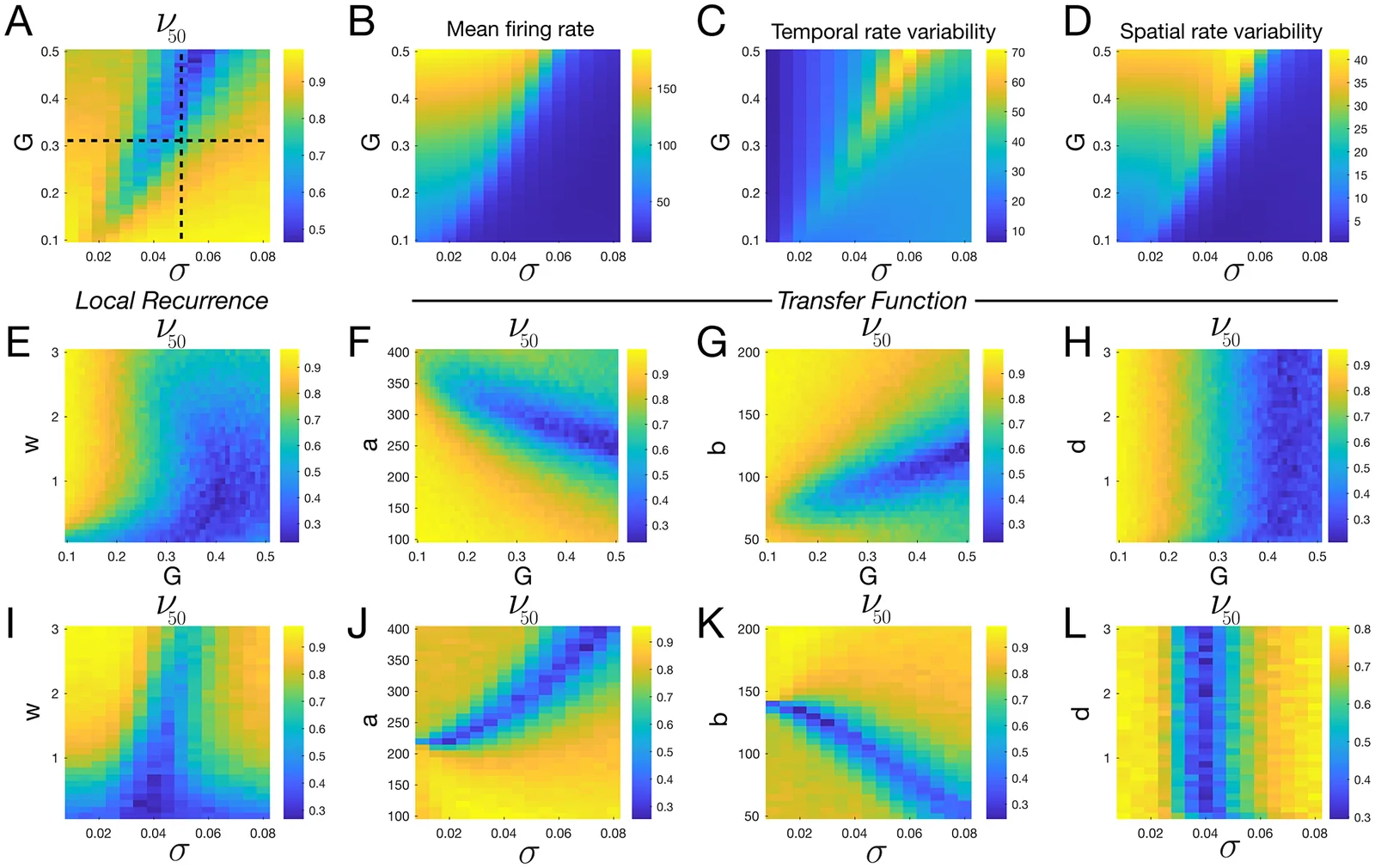
Regimes of faster or slower FCD. **(A)** Global coupling parameter *G* (scales cortical excitability) and noise amplitude σ (independent gaussian noise supplied to each brain region) collectively modulate FC speed (heat map indicates median speed $\nu_{50}$ distribution). A level neither too low, nor too large of baseline drive noise σ is necessary to arrive at the blue region corresponding to a regime of slower FC dynamics, suggestive of stochastic resonance. **(B–D)** The mean firing rate, temporal rate variability (mean of the standard deviation of each ROI’s firing rate) and spatial rate variability (standard deviation of the mean of each ROIs firing rate) as a function of *G* and σ. The slower FCD regime corresponds to a regime of dynamic bistability in the firing rate of cortical regimes, akin to spatially distributed transitions between *up* and *down* states [15,37]. **(E–H)** Median FCD speed as a function of local model parameters and *G*. **(I–L)** FCD speed as a function of local model parameters and σ.

The parameter sweep revealed that *G* and σ exert the strongest influence on FCD speed, giving rise to distinct dynamical regimes (Fig 2A). In the *G*–σ plane, a wedge-shaped region emerged where FCD speed—here tracked by the obtained median value $\nu_{50}$—was markedly reduced, suggesting the presence of a critical transition zone. Notably, entry into this regime required a finite level of stochastic input, consistent with a form of stochastic resonance [44]. The low-speed wedge coincided with elevated temporal rate variability (mean standard deviation of firing rates; Fig 2C), whereas its lower boundary aligned with a rate instability that produced increased spatial rate variability across regions (Fig 2B). Our results are consistent with previous parametric studies of the Wong–Wang whole-brain model, which identified two rate boundaries at higher global coupling: a lower threshold is associated with the critical *ignition* line (*G*_-_), at which some regions, due to their dense neighborhood, are able to self-sustain themselves in a “up” state with large firing rate, even in the absence of strong external drive; and an upper *flaring* threshold marking a rate instability (*G*_+_) beyond which all regions saturate in their high firing rate regime [15,37].

Across all examined parameters, the nonlinear nature of the MFM generated sharp phase transition boundaries, underscoring its sensitivity to small perturbations in parameter state (Fig 2E–2L). These findings indicate that modest parameter changes can reorganize the dynamical regime of large-scale brain networks.

### Arousal-Like global modulatory drive in the MFM

Could fluctuations in arousal constitute the biological mechanism driving small parameter changes that traverse critical regimes and thereby reorganize large-scale dynamics? We hypothesized that slow, endogenous variations in arousal modulate key model parameters such as global coupling (*G*) and noise amplitude (σ)—thereby steering the system through different regions of the parameter space identified in the prior sweep.

To test this, we introduced an explicit arousal variable evolving as a smooth, time-dependent signal that parametrically influenced model parameters. This variable serves as a proxy for diffuse, global arousal inputs—such as those arising from brainstem and neuromodulatory systems, including the locus coeruleus—whose widespread projections influence large-scale brain dynamics. We emphasize that this formulation is intended as a simplified modeling abstraction of arousal-related neuromodulatory influences, capturing their aggregate, slow effects on model parameters rather than providing a direct or one-to-one representation of physiological arousal. In what follows, we continue to refer to this mechanism as “arousal” for simplicity.

For generality, we modeled this arousal signal as a truncated Ornstein**–**Uhlenbeck process, defined by a baseline value, mean-reversion rate, and stochastic drive. The baseline tone reflects empirical observations from pupil diameter and locus coeruleus firing, which indicate distinct tonic and phasic neuromodulatory modes [45].

This formulation transforms the MFM from autonomous to nonautonomous—i.e., from static to time-dependent—, allowing arousal to act as a global control parameter that continuously modifies the system’s DWP and thereby, its dynamical landscape. We then assessed whether arousal-driven modulation reproduces the temporal structure and variability of FCD observed in human fMRI data, and how it compares to the autonomous model. To probe which portions of the FCD and FCD-speed distributions are most influenced by neuromodulatory control, we parametrized FCD and FCD-speed distributions using percentiles ($5^{th}$, $25^{th}$, $50^{th}$, $75^{th}$, $95^{th}$) and used these as fitting objectives in a genetic algorithm (GA) [46] (see Methods).

**Fig 3:**
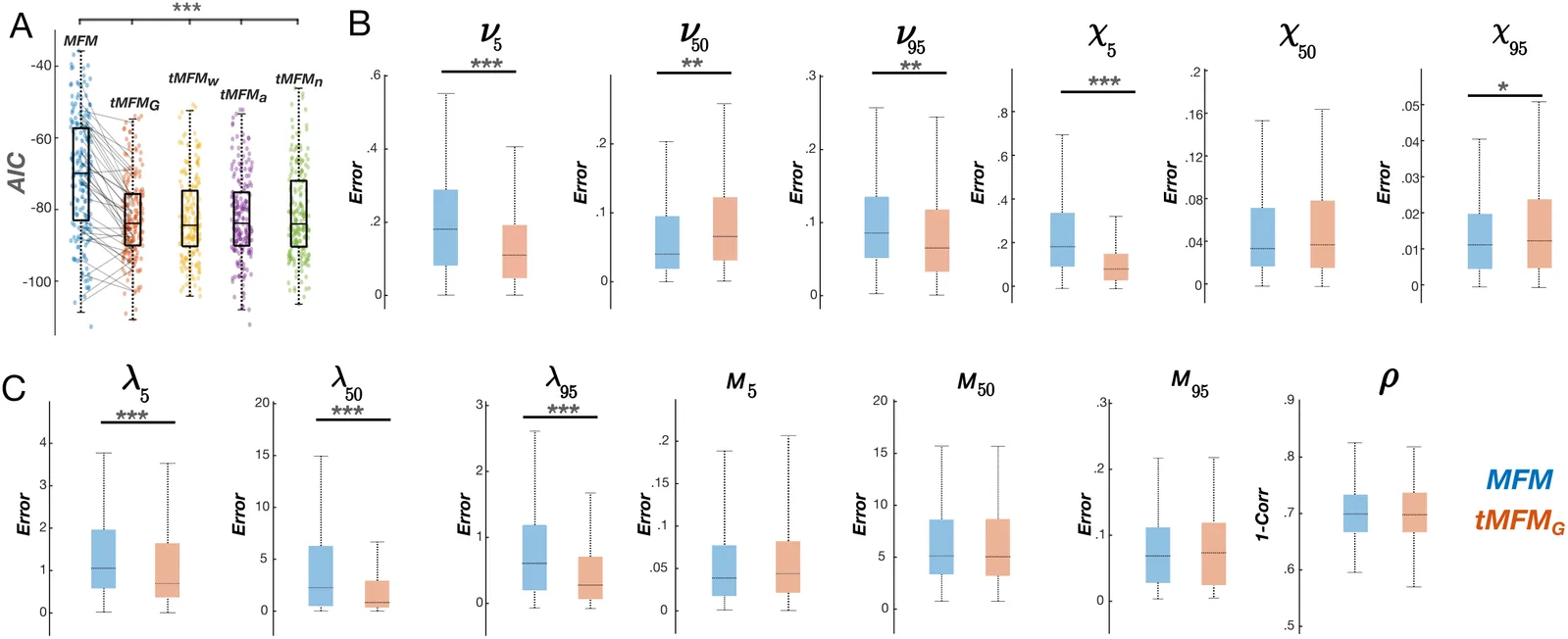
Nonautonomous models outperform autonomous models at explaining detailed FCD features. **(A)** Comparison of 5 models (1 autonomous and 4 nonautonomous) in their ability to fit a 10-dimensional vector composed of FCD and FC Speed features ($5^{th},25^{th},50^{th},75^{th},95^{th}$ percentiles of FCD matrix χ and FCD speed ν distributions) derived from 200 HCP recordings. Akaike Information Criteria (AIC) penalizes for model complexity **(B)** Box-plots indicating the error incurred by each model for fitting targets (FCD Speed ν, FCD Distribution χ) and **(C)** prediction targets (ρ,λ, *M*) for the entire dataset consisting of 200 recordings. Results suggest robust statistical fitting by nonautonomous as compared to autonomous models. The data underlying this Figure can be found in S1 Data.

**Fig 4:**
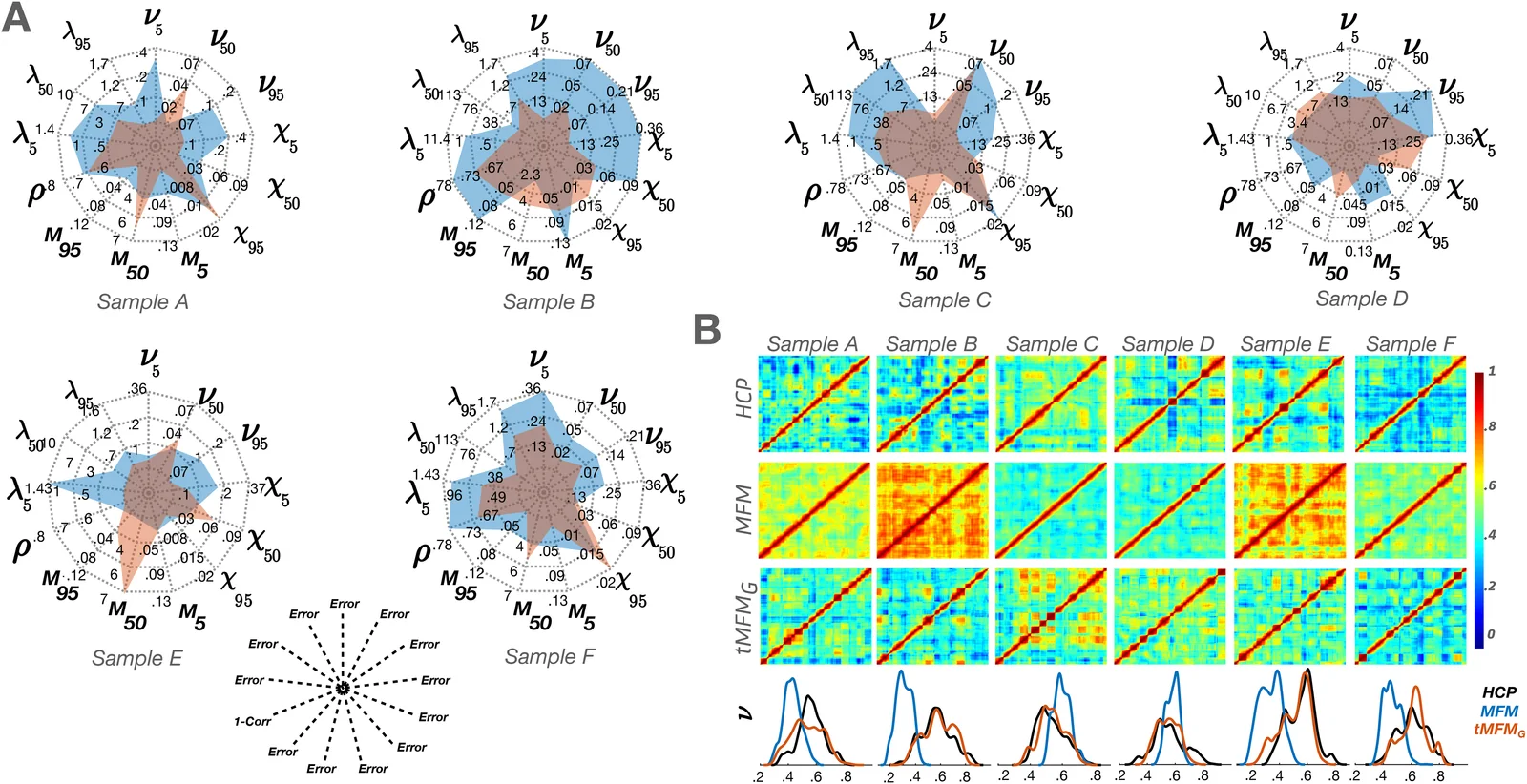
Sample fits: (A) spider plot comparing the ability of the *MFM* (autonomous, blue) and $tMFM_{G}$ (nonautonomous, red) at capturing the $5^{th},50^{th}$ and $95^{th}$ percentiles of FCD (χ) and FC speed (ν) distributions for 6 sample recordings. Additionally, the spider plot indicates how the two models predict match to static FC (ρ, computed as correlation distance) and windowed SC-FC correlations (λ) and Meta-Connectivity (*M*) that were not used as direct fitting targets. **(B)**. FCD recurrence matrices for the same six representative sample recordings and the corresponding best fits offered by the MFM and ${\text{tMFM}}_{G}$ models. In the bottom row, comparison between empirical and model FCD speed distributions for the same 6 recordings.

Comparing autonomous and nonautonomous models across 200 recordings (see summary statistics in Fig 3 and details for five representative subjects in Fig 4), we found that incorporating arousal-driven modulations of model parameters significantly improves model fit, even when penalizing for the additional parameters introduced by the Ornstein–Uhlenbeck process (see S1 Fig for absolute errors). Focusing on the ${\text{tMFM}}_{G}$ model (in which *G* is under neuromodulatory control), most of this improvement stems from its ability to accurately capture the tails of the FCD fluidity χ and speed ν distributions—as illustrated by the example distributions shown at the bottom of Fig 4B—and in particular the left tail ($5^{th}$ percentile). This finding is consistent with our hypothesis that neuromodulatory inputs selectively enhance slow dynamical regimes (Fig 1). At the same time, we observed that the ${\text{tMFM}}_{G}$ model also markedly improves the representation of the right tail of the FCD-speed distribution ($95^{th}$ percentile of ν), suggesting the occurrence of abrupt crossings of critical transition boundaries that are absent in the autonomous model (Figs 3C and 4A).

To further assess heterogeneity of model performance across subjects, we performed unsupervised clustering (k-means) on relative Akaike Information Criteria (AIC) values, identifying four robust clusters. In three clusters, the tMFM class consistently outperformed the static MFM to varying degrees, whereas a fourth cluster corresponded to cases where all models performed similarly (S2 and S3 Figs). Examination of representative subjects revealed that this latter group was associated with atypical dynamics (high FCD χ, low FCD speed ν), suggestive of noisy or low-quality recordings (S4 and S6 Figs). Excluding this category, the results indicate that tMFM models provide a better account of the data, with minimal differences across their variants in terms of AIC.

### Predictive power of arousal fluctuations

A good model should not only fit the data it was trained on but also predict independent features that were never part of the fitting process. Adopting this philosophy, we next evaluated how well the nonautonomous, arousal-driven models generalize to other spatiotemporal properties of ongoing brain activity. Specifically, we examined their ability to predict: (i) static functional connectivity (sFC); (ii) the temporal dynamics of structure–function coupling (SC–FC coupling); and (iii) Meta-Connectivity (MC), which captures the covariance among time-varying FC links (Table 1).

**Table 1:** Summary of features used for model fitting and out-of-sample validation. All distributions are summarized as the $5^{th},25^{th},50^{th},75^{th}$ and $95^{th}$ percentiles. See Methods for detailed mathematical formulae.

Category	Feature	Description
Model fitting	FCD (χ)	Distribution of functional connectivity dynamics matrix
	FCD speed (ν)	Distribution of step lengths between subsequent, nonoverlapping FC frames ($1-corr[FC_{t},FC_{t+1}]$)
Prediction	Meta-connectivity (M)	Distribution of time-varying correlation between all edges
	SC–FC coupling (λ)	Distribution of time-varying correlation between SC and FC
	Static FC (ρ)	Correlation between empirical and simulated time-averaged FC

Static FC has historically served as the primary benchmark for whole-brain modeling, with several studies showing that even linear or purely statistical models can capture much of its variance [47–49]. Consistent with this, both the autonomous (MFM) and nonautonomous mean-field models (tMFM) reproduced the broad structure of empirical FC, with no significant difference in the correlation ρ between simulated and empirical sFC matrices (Fig 3C).

**Fig 5:**
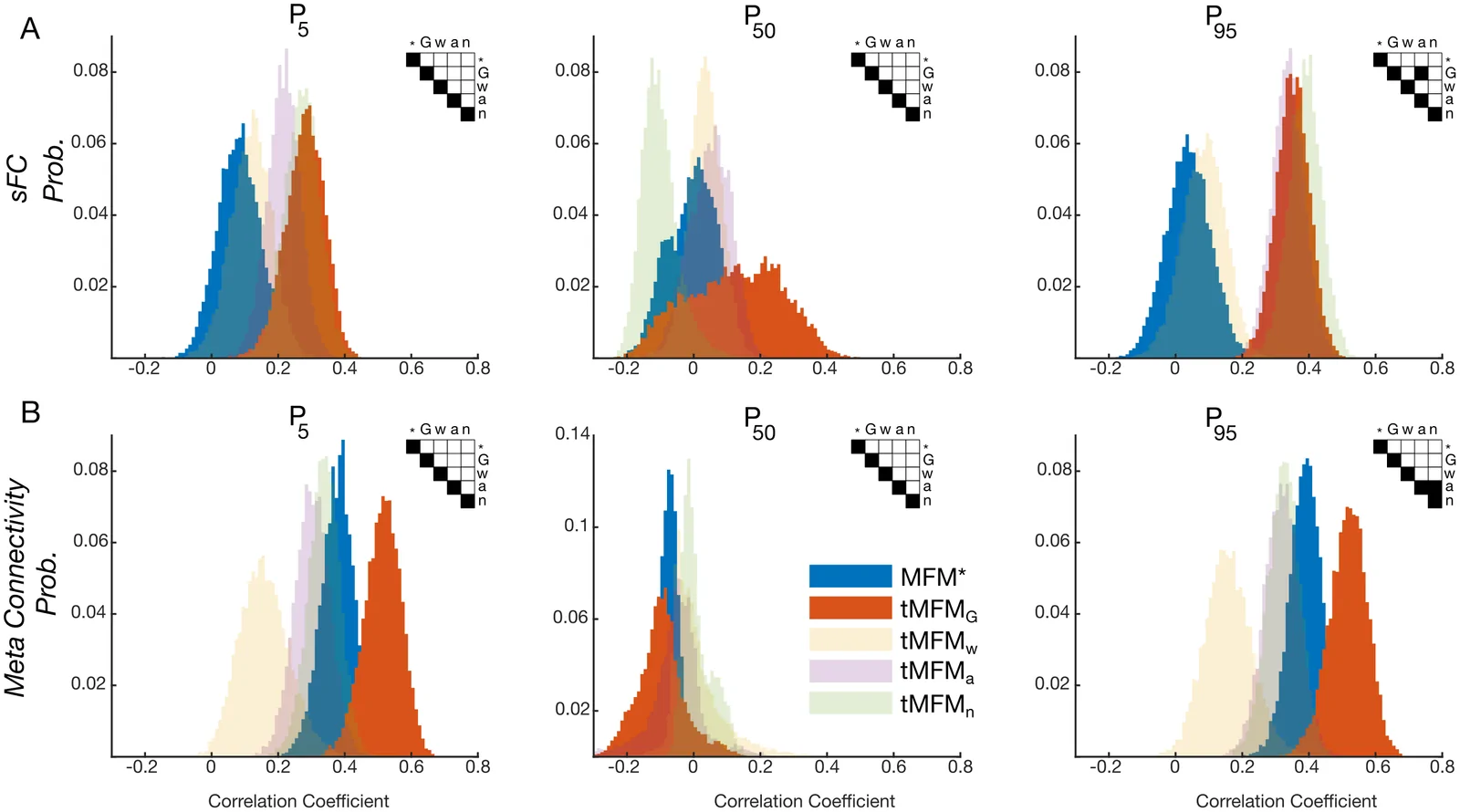
Nonautonomous models outperform autonomous models in novel feature prediction. We assessed how well the different models reproduced empirical FCD features that were not explicitly fitted during model optimization. We report distributions of bootstrapped correlations between simulated and empirical features, computed for percentiles of the single-subject distributions of **(A)** static FC matrix entries and **(B)** Meta-Connectivity entries. Checkered insets indicate significant differences between the correlations achieved by different model types (black = not significant). nonautonomous models, and in particular the ${\text{tMFM}}_{G}$, consistently yield higher correlations.

To detect subtler effects beyond this gross matrix similarity, we further examined how accurately the models captured the range of individual pairwise sFC weights at the single-subject level. For each subject, we extracted the distribution of sFC matrix entries and computed, across recordings, the correlation between empirical and simulated values of the $5^{th}$, $50^{th}$, and $95^{th}$ percentiles of these distributions. This analysis allows us to assess how precisely the fitted models capture inter-subject differences in the range of sFC values. Using bootstrap resampling (see Methods) with replacement to estimate correlations and confidence intervals, we found that models incorporating arousal-dependent modulation of global excitability markedly improved the prediction of sFC percentiles. This result suggests that abrupt, arousal-induced transitions contribute substantially to shaping mean FC patterns (see Fig 5A for bootstrap distributions).

We next examined the similarity between structural connectivity (SC) and individual frames of the FCD reconfiguration stream. This SC–FC correlation is known to fluctuate over time, reflecting alternations between epochs in which time-resolved FC is more or less constrained by the underlying structural architecture [50,51]. For this feature, both the autonomous (MFM) and excitation-modulated (${\text{tMFM}}_{G}$) models overestimated the overall level of SC–FC correlation (λ), yielding average values of approximately −.004,0.229,0.05 and −0.0131,0.0132,0.0396, respectively, compared with the empirical mean of −0.142,0.0082,0.0305 for the $5^{th},50^{th},95^{th}$ percentiles. Despite this offset, the nonautonomous model captured the quantiles of the λ distribution with lower percent error, providing a better representation of the tails than the median (Figs 3C and 4A). This improvement suggests that critical bifurcations—enabled by arousal-driven fluctuations—are necessary to reproduce the intermittent decoupling between structure and function observed in human data.

Finally, we turned to MC [5,11], a form of edge-based functional connectivity [36] that quantifies the co-fluctuations of pairwise FC links. MC provides a static description of coordinated temporal fluctuations in FC link strengths, in much the same way that classical sFC provides a static description of coordinated temporal fluctuations in regional node activity. The structure of MC, and in particular its modular organization [5,36], reveals the existence of sets of links—and hence functional subnetworks—that coherently “pop in” and “out” during the FCD stream. This indicates that FCD is organized not only temporally, but also spatiotemporally. Just as for sFC, we assessed how well the models reproduced the distribution of MC entries at the single-subject level, using distribution percentiles as prediction targets rather than quantities directly fitted during model optimization. Analogously to Fig 5A, we report bootstrapped correlations between the $5^{th},50^{th}$, and $95^{th}$ percentiles of matched simulated and empirical MC-entry distributions (Fig 5B). Once again, the ${\text{tMFM}}_{G}$—driven by global fluctuations in cortical excitability (*G*)—significantly outperformed both the autonomous and alternative nonautonomous models in accounting for empirical MC statistics. Specifically, the improvement is most pronounced for the $5^{th}$ and $95^{th}$ percentile, corresponding to the lower and upper tail of the MC-entry distribution, which also includes rare negative MC values. This enhanced ability to capture the left tail is particularly important, as the emergence of “viscosity”—that is, the appearance of an increased number of negative MC entries—has been associated with pathological progression in neurodegenerative diseases such as Alzheimer’s disease [11,12].

We note that, in the preceding analysis, both the sFC and MC were extracted after performing a global signal regression (GSR). This choice is motivated by the fact that in the *tMFM*, a neuromodulation of model parameters is implemented as a uniform scaling of the parameter across all the nodes, which has the tendency of biasing positive correlations due to common inputs (S7 Fig). Applying GSR mitigates this common-input effect, enabling a more balanced comparison of correlation structure. Importantly, however, even in the absence of GSR—where absolute errors are larger—the bootstrap correlation analysis (which is insensitive to absolute scaling) continues to demonstrate the superiority of the $tMFM_{G}$ (compare Fig 5B with S8 Fig). This indicates that the improved performance of $tMFM_{G}$ is not solely driven by global correlation biases, but reflects its ability to capture meaningful structure in the data. Further, we assessed whether the model’s ability to reproduce off-target features arises trivially from fitting the target metrics by analyzing their statistical relationships. The $tMFM_{G}$ captures both individual features and their inter-feature correlation structure significantly better than the baseline MFM, indicating that these relationships are nontrivial (S9 Fig).

Taken together, these results demonstrate that arousal-driven modulation improves not only goodness of fit, but also predictive generalization across multiple spatiotemporal scales of brain dynamics.

### Neuromodulatory inputs induce working point excursions across critical phase transition boundaries

The superior performance of the arousal-driven model can be traced to how arousal fluctuations navigate the system’s phase space. The MFM exhibits distinct regimes demarcated by slower (blue) and faster (yellow) speed of FCD reconfiguration (Fig 2A). In the arousal-modulated model, fluctuations in the working point allow the system to roam back and forth across these critical boundaries, producing abrupt dynamical transients. This capacity for controlled excursions between regimes appears to underlie the enhanced temporal flexibility captured by the model.

Taken together, the fitting and predictive analyses converge on a simple conclusion: slow, global fluctuations in cortical excitability (captured by the parameter (*G*) provide a parsimonious account of the temporal organization of FCD. To examine this mechanism more directly, we compared the DWPs inferred from model fits of autonomous and nonautonomous formulations to empirical resting-state recordings. In the autonomous models, fitting yields a single, time-invariant DWP defined by the optimal values of (*G*) and (σ). In contrast, the nonautonomous tMFM models allow selected parameters to fluctuate over time; for each such parameter we estimated a baseline level, a characteristic timescale, and a volatility, which together define both the range of variation and the corresponding occupancy distribution. We focused in particular on the inferred fluctuation range of the global coupling parameter (*G*) in the fitted $tMFM_{G}$ models.

**Fig 6:**
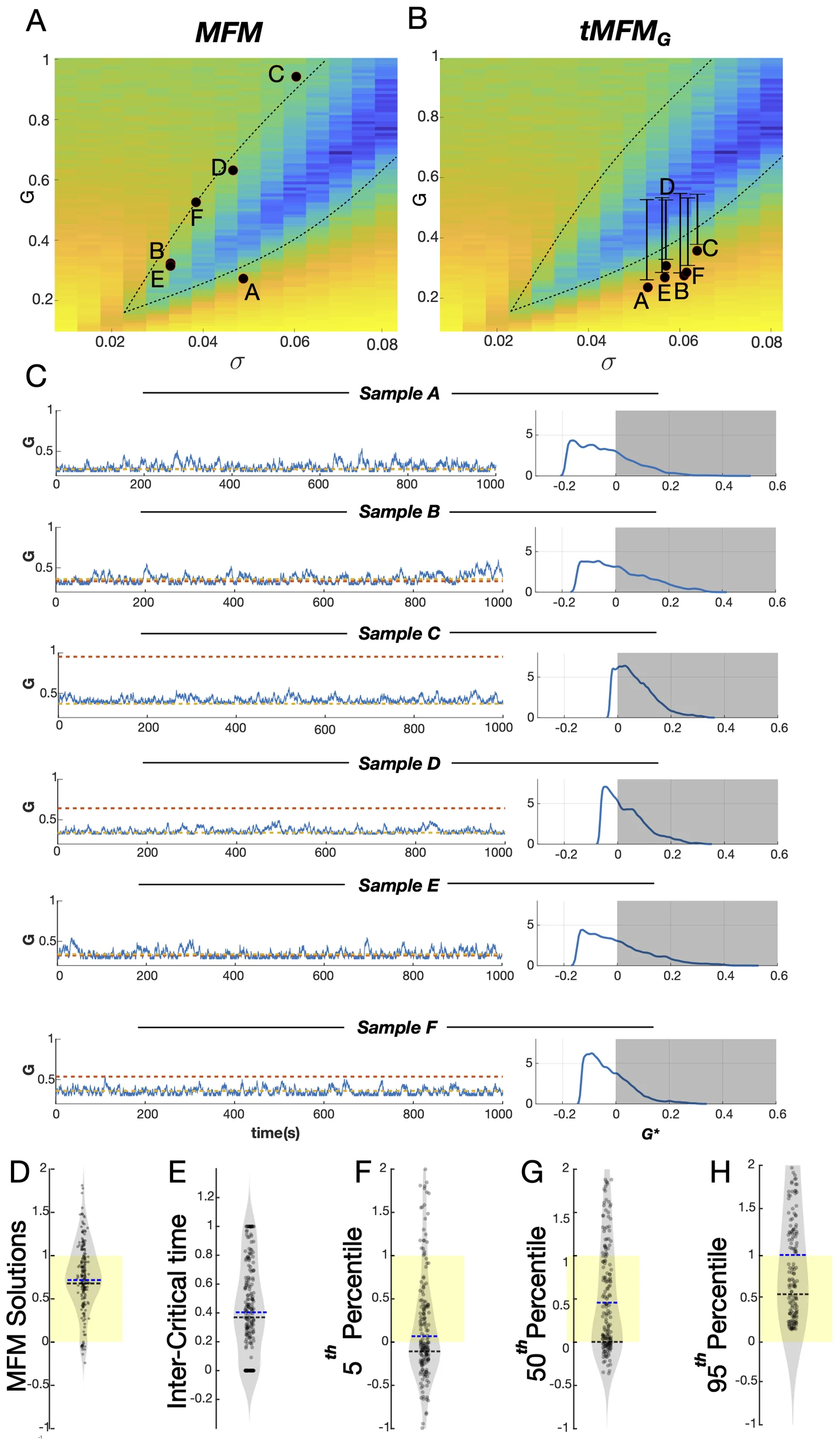
From the critical point to the critical roaming hypothesis. **(A)** Inferred working points for the MFM (autonomous) for six representative subjects; best fit DWPs lie close to the critical flaring rate *G*_+_ instability. As in Fig 2A–2D, *G* scales the strength of inter-regional connections while σ is the amplitude of baseline drive noise supplied to each node. Heatmap color indicates the median FCD speed ($<\nu_{50}>$). **(B)**. For the ${\text{tMFM}}_{G}$ (nonautonomous model), a range of possible values that a fluctuating G* can assume is fitted instead. The inferred ranges indicate that the DWP has a baseline level below the critical ignition boundary *G*_-_ and makes transient excursions beyond it. **(C)**. (Left) Time series of *G* for the six representative subjects relative to the MFM solution (red) and critical ignition boundary (yellow). (Right) The dwell time distributions of *G*, i.e., cortical excitability *G* scaled relative to the Lower (*G*_-_) and Upper critical boundaries (*G*_+_), with $G^{*}=\frac{G-G_{-}}{G_{+}-G_{-}}$. Shaded region lies within the slow speed intercritical range. **(D)**. The range of solutions for the autonomous MFM across 200 recordings **(E)** The amount of time spent within the slow regime as a fraction of total area under curve. **(F–H)** The distribution of the $5^{th}$,$50^{th}$ and $95^{th}$ percentiles of the normalized $G^{*}$. The average distribution of the percentiles indicates the dynamic range explored by ${\text{tMFM}}_{G}$. The dashed blue lines indicate the distribution medians and black dashed line the distribution modes. Overall, the fitting of nonautonomous models suggest that the system spends a substantial part of its time roaming between the ignition and flaring critical transitions. The data underlying this Figure can be found in S1 Data.

As shown in Fig 6A, the DWPs inferred for MFM of the six representative subjects in Fig 4 cluster tightly near the critical boundary associated with the model’s “flaring” rate instability, ($G_{+}(\sigma )$). This pattern generalizes to the full dataset of 200 recordings (Fig 6D), where the majority of inferred solutions likewise lie in close proximity to this instability line. Together, these results lend strong support to the longstanding hypothesis that large-scale brain dynamics operate near critical transitions [19–21], at least when described by autonomous whole-brain models.

In contrast, we show in Fig 6B the range between the fitted baseline minimum and maximum values of *G* in the non autonomous ${\text{tMFM}}_{G}$ for the same six subjects of Fig 6A. In most solutions, the baseline excitability was at values slightly below the ignition critical boundary (Fig 6B, 6C) (*G*_−_). However, as an effect of parameter fluctuations in time, the system frequently entered the critical zone between the ignition and the flaring lines, spending a substantial fraction of its time within the slow regime (between *G*_−_ and *G*_+_) (Fig 6C, right).

To quantify more precisely and systematically, across the full set of fitted sessions, the range of DWPs transiently explored by the system and their relation to the lower ignition and upper flaring critical lines, we introduced a normalized index $G^{*}=\frac{G-G_{-}(\sigma )}{G_{+}(\sigma )-G_{-}(\sigma )}$. By construction, $G^{*}<0$ when *G* is subcritical with respect to the ignition point $G_{-}(\sigma )$ at the fitted value of σ; $0\leq G^{*}\leq 1$ when the system roams within the slow intercritical regime between the ignition and flaring points $G_{+}(\sigma )$; and $G^{*}>1$ when it lies beyond the flaring transition. These critical boundaries were defined in a data-driven fashion by performing k-means clustering of the phase diagram spanning FCD, FCD speed, mean firing rates, and spatio-temporal rate variability features (S10 and S11 Figs). Fig 6E shows the fraction of time spent in the slow intercritical regime, which amounts to ~40%. Fig 6F–6H instead report the quantiles of the single-session distributions of $G^{*}$.

Baseline cortical excitability, tracked by the 5th percentile of the single-session distribution of $G^{*}$, had a median value close to 0, i.e., near the ignition point *G*_-_ (Fig 6F), while the upper range of excitability, tracked by the 95th percentile of the distribution of $G^{*}$, had a median value close to 1, i.e., near the flaring point *G*_+_ (Fig 6H). Despite variability across recordings, the tails of the normalized *G* distributions thus consistently indicated subcritical baselines punctuated by frequent excursions beyond the lower ignition critical line. A smaller subset of solutions exhibited crossings of both the lower ignition and upper flaring boundaries, or of the upper boundary alone, reflecting transient transitions into fast, high-excitability regimes (Fig 6F–6H and S12 Fig).

Taken together, these results suggest that, in contrast with the critical point hypothesis—according to which the DWP remains close to the flaring rate instability, as implied by the autonomous best-fit models—arousal-driven modulations enable the brain to dynamically explore the entire range between the ignition and flaring lines, intermittently departing from a subcritical baseline below ignition. We refer to this alternative scenario, supported by the nonautonomous model fits, as the *critical roaming hypothesis*.

## Discussion

Spontaneous brain activity is richly structured in time, yet the dynamical principles governing these fluctuations have remained elusive. Here we show that slow, global modulations of cortical excitability—an interpretable proxy for neuromodulatory arousal—provide a parsimonious explanation for the heavy-tailed, intermittently reorganizing structure of FCD. By directly contrasting conventional MFM with explicitly nonautonomous, arousal-driven variants, we demonstrate that many hallmark features of ongoing FC—fat-tailed step-length distributions, alternating epochs of persistence and rapid reconfiguration (Fig 2), and additional out-of-sample signatures such as SC–FC coupling, fit to static FC and MC (Fig 5)—emerge only when the model’s DWP is allowed to drift over time. In the autonomous case, reconfigurations require the system to hover near intrinsic bifurcations; the nonautonomous model naturally traverses these transitions, greatly expanding the repertoire of accessible large-scale network states (Fig 6).

A key conceptual advance enabling this result is our explicit focus on the sequential organization of FCD. Building on previous work quantifying FC variability, we introduce FCD Speed as a measure of the stepwise progression of the system through FC space [4,5,11]. Unlike statistics that summarize FCD variability alone [52,53], FCD Speed captures the stochastic, random-walk–like structure of FC trajectories and their occasional large “leaps” [4]. These heavy-tailed excursions turn out to be crucial: improvements in model performance arise almost entirely from the tails of the FCD and FCD-Speed distributions, not their means, which both model families already reproduce well (Fig 3). This highlights that meaningful dynamical events—those that reshape whole-brain FC—are rare, abrupt, and deeply informative about underlying mechanisms.

The presence of such intermittent, large-scale reconfiguration events naturally raises the question of where the brain operates relative to critical boundaries. Criticality has long been proposed as an organizing principle of neural dynamics, yet its functional role remains debated. Emerging evidence suggests that the brain does not maintain a fixed proximity to a critical point; rather, it approaches or retreats from criticality depending on behavioral and cognitive demands. Simple tasks may even be hindered by critical dynamics, whereas demanding tasks benefit from them [54], implying that the “distance to criticality” is itself a tunable control variable. Arousal and vigilance are known to shift this distance [55], and resting-state fMRI—particularly in eyes-closed conditions—captures drifting mixtures of wakefulness and light sleep [56]. This body of work suggests that the brain’s DWP is inherently nonstationary, wandering across a wide swath of state space, such that small fluctuations in arousal can produce disproportionately large changes in FC. This may explain why linear models approximate static FC well [47–49] (S13 Fig) but systematically fail to capture the temporal richness of time-resolved FC.

Our fitted simulations reveal how such wandering arises: slow fluctuations in a global excitability-like parameter (captured by the scaling term (*G*)) repeatedly move the system toward and away from rate-instability transitions (Fig 6). Although several model parameters can, in principle, induce critical transitions, perturbation analyses consistently identified (*G*) as the dominant determinant of FCD temporal structure. Biologically, this resonates with the diffuse ascending projections of major neuromodulatory systems, which act as global gain controllers and are well positioned to modulate large-scale excitability [25,26,57]. Nonetheless, neuromodulatory influences are not isolated control knobs but coordinated, low-dimensional patterns of receptor- and state-dependent action. The strong explanatory power of (*G*) therefore likely reflects a projection of this low-dimensional biological manifold onto model space, rather than the dominance of any single biophysical mechanism. Importantly, the nonlinear structure of the model endows it with a rich phase space, within which modulations of parameters such as *G* or noise amplitude (σ) can induce transitions across regimes and give rise to phenomena such as stochastic resonance. As a result, FCD can exhibit nonmonotonic (U-shaped) dependencies—for instance in FCD speed—reflecting an optimal intermediate regime between overly stable and overly labile dynamics (for example, see Fig 2F). Such behavior is consistent with empirically observed reorganizations of brain activity and resonates with classical principles such as the Yerkes–Dodson law [58], highlighting how complex, arousal-like effects can emerge from generic nonlinear dynamics. Moreover, incorporating realistic spatial gradients of neuromodulatory receptor densities—known to be systematic across cortex [40,59]—may further enhance the model’s ability to capture fine-grained spatial structure in MC, which we considered here only in distributional form.

Our findings also carry important implications for the fMRI community, which has long debated the extent to which resting-state FCD is shaped—or contaminated—by arousal-related fluctuations [27,60]. The present framework offers a principled, dynamical-systems–based method for quantifying these contributions. In particular, it provides a mechanistic counterpart to the distinction proposed by Laumann and colleagues [27], who separated neural contributions to FCD into components related to spontaneous cognition versus arousal. Within our modeling scheme, this dichotomy maps naturally onto autonomous versus nonautonomous dynamics: the former capturing intrinsic, cognition-related fluctuations, and the latter reflecting slow, state-dependent modulations driven by arousal systems.

Several additional observations warrant consideration. First, we turn to the role of negative correlations (anti-correlations) in FCD, which are increasingly recognized as functionally meaningful [61]. A limitation of our framework is that neuromodulatory effects are modeled as uniform modulations of the model parameters, which biases the system toward increased positive correlations due to shared fluctuations across regions. Applying GSR partially mitigates this effect and introduces negative values in the tail of the MC distribution (Figs 3, 4); however, these anti-correlations cannot be unambiguously attributed to intrinsic dynamics, as GSR itself is known to induce such effects [62]. Notably, the static MFM already exhibits negative MC tails even in the absence of GSR (see S7 Fig), suggesting that at least part of this phenomenon may arise from the underlying nonlinear dynamics. Disentangling these contributions represents an important direction for future work. One potential avenue to mitigate the need for GSR, and reduce common-input effects, is to introduce parametric heterogeneity—for example, by incorporating spatial gradients of receptor densities.

Secondly, while the predominant dynamical motif across participants consisted of fast-regime trajectories interrupted by intermittent excursions into the slow regime, we also observed substantial inter-individual heterogeneity. In a subset of individuals, trajectories originated within the slow regime and periodically crossed rate-instability boundaries, suggesting that different brains may occupy systematically distinct positions along an arousal–excitability manifold. Such alternative dynamical pathways may reflect stable differences in baseline arousal, neuromodulatory responsiveness, or cognitive style, and may ultimately map onto meaningful behavioral, age-related, or clinical phenotypes [52,63–65]. Converging evidence from neuropathology, imaging, and clinical studies indicates that neurodegenerative disorders—most notably Alzheimer’s disease (AD)—are characterized by early and progressive disruption of ascending neuromodulatory systems. Degeneration of basal forebrain cholinergic nuclei is a hallmark of AD and underlies the long-standing cholinergic hypothesis, with loss of cholinergic neurons and cortical innervation closely tracking cognitive decline [66,67]. Similarly, the noradrenergic locus coeruleus shows pronounced vulnerability, often degenerating prior to overt cortical pathology and clinical symptoms [68]. Because these systems exert diffuse control over local cortical parameters, their degeneration is expected to constrain the brain’s ability to dynamically traverse critical regimes of network dynamics. Within the framework developed here, such neuromodulatory loss would restrict excursions across dynamical phase boundaries, narrowing the accessible repertoire of functional network states. This may provide a parsimonious mechanistic account of the reduced dynamical fluidity [69], impaired FCD [11], and diminished metastability [70] consistently reported in AD, and suggests that neuromodulatory decline contributes to cognitive impairment by producing a fundamental impoverishment of large-scale brain dynamics.

We offer potential customizations of the pipeline proposed here. First, although our implementation relied on the bistable Wong–Wang neural mass—a widely used model for large-scale fMRI dynamics [15,40,41]—the broader framework we introduce is not tied to this specific formulation. Oscillatory mechanisms, which are central to EEG, MEG, and LFP signals, could be readily incorporated using Stuart–Landau oscillators or next-generation neural mass models such as the Montbrió mean-field reduction [71,72]. Extending the framework into these oscillatory regimes may offer a principled route for unifying fast electrophysiological rhythms with slow arousal fluctuations [30,73]. Similarly, node dynamics could be extended to include additional targets of neuromodulatory action. In particular, mean-field models incorporating spike-frequency adaptation—known to be modulated by acetylcholine—provide a biologically grounded mechanism for shaping large-scale dynamics, including the emergence of Up–Down state transitions characteristic of NREM sleep. Such extensions could be implemented using mean-field reductions of models like the Adaptive Exponential Integrate-and-Fire model, enabling a more mechanistic investigation of how neuromodulation influences FCD [74,75].

Second, we adopted a GA for model fitting because it robustly accommodates noisy, computationally expensive simulations and multi-objective optimization without requiring gradient information [46]. This makes it particularly well suited to whole-brain models, whose parameter spaces are often highly nonlinear and characterized by sharp dynamical transitions. By treating the model as a black box, this approach avoids explicit assumptions about likelihood functions or the need for specialized training datasets. Recent work has explored simulation-based inference [76,77] for parameter estimation in whole-brain models, and systematic comparisons of these complementary approaches—in terms of performance, scalability, and interpretability—represent an important direction for future research. Finally, the whole-brain modeling community has increasingly leveraged PET-derived neurotransmitter maps to incorporate spatial heterogeneity in neuromodulatory influences [78–82]. When combined with the temporal framework introduced here, such spatially informed approaches could provide unprecedented insight into how distinct neuromodulatory systems shape the spatiotemporal organization of large-scale network dynamics, enabling a rich repertoire of functional configurations to emerge from a fixed structural scaffold. The temporal dynamics of arousal could be further informed by diverse data streams that serve as arousal proxies such as pupil diameter and electrophysiological markers of arousal [30,83]. These multimodal measures provide a natural testbed for evaluating and refining the mechanistic hypotheses proposed here, and the present model—while intentionally coarse—can be readily augmented to incorporate such information in future extensions.

Together, our findings offer a unified framework in which neuromodulation shapes large-scale brain dynamics by continuously steering the system across inter-critical regions of state space. Rather than treating resting-state activity as noise around a fixed operating point, our results support a view of the brain as an adaptive dynamical system whose working point fluidly evolves on slow timescales, enabling it to flexibly explore, sample, and reorganize its FC landscape. This perspective provides a foundation for mechanistically linking arousal, critical dynamics, and spontaneous cognition—and for understanding how their disruption contributes to aging, neuropsychiatric conditions, and altered states of consciousness.

## Methods

### Dataset

The dataset analyzed here corresponds to the same subset of the Human Connectome Project (HCP) used in prior work on test–retest reliability [84]. It includes resting-state fMRI recordings from 100 healthy adults collected by the HCP WU–Minn Consortium. Each participant completed two eyes-open resting scans on separate days while fixating a central cross (200 recordings in all). Functional data were acquired on a 3T Siemens Connectome Skyra using a multiband gradient-echo EPI sequence (2-mm isotropic voxels; TR, 720 ms; TE, 33.1 ms; multiband factor, 8), yielding 1,200 volumes per run (14 min 24 s). High-resolution T1- and T2-weighted images (0.7-mm isotropic) accompanied each session. For all analyses, we used the version of this dataset parcellated into 89 anatomical regions using the AAL atlas.

### FC, FCD, and FCD Speed

For both empirical and simulated BOLD data, FCD was computed using a standard sliding-window approach. Each time series was segmented into overlapping windows of 60 s, advanced in 2-s steps (58-s overlap), following [15]. FC was estimated within each window, and pairwise correlations between the upper-triangular elements of all windowed FC matrices were assembled into the FCD matrix:


$$\begin{array}{c} \chi (t_{1},t_{2})=corr(UpperTr[FC(t_{1}),UpperTr[FC(t_{2})]] \end{array}$$ (1)

FCD speed was quantified as the rate of change between FCs derived from successive nonoverlapping windows:


$$\begin{array}{c} \nu =1-corr(FC(n),FC(n+1)) \end{array}$$ (2)

The distance metric used above is the standard Euclidean distance, although FCD matrices are symmetric positive definite (SPD) and therefore lie on a curved manifold. We retain the Euclidean measure for simplicity and to facilitate comparison with prior studies that adopt the same approach [4,85,86]. However, we note that, in principle, alternative distance measures that respect the geometry of the SPD manifold could also be employed.

Further, to increase sampling density for relatively short recordings, speeds were computed using window lengths between 55 *s* and 65 *s* and then pooled. To further assess if there is a significant impact of spurious correlations due to limited window sizes, we applied random matrix theory–based denoising to the FC streams by filtering eigenvalues below the Marchenko–Pastur threshold ($\frac{N_{ROI}}{WinSize}$) and reconstructing the FC matrices from the retained components. We then recomputed FCD speed from the filtered FCs and compared it to the original estimates. The high correspondence between the two (correlation > 0.9 across subjects) indicates that noise-related effects are minimal and do not materially affect our analysis (S14 and S15 Figs).

sFC (ρ) was computed as the Pearson correlation between the time series of all pairs of ROIs over the entire recording. Time-resolved SC–FC coupling (λ) was obtained by correlating each instantaneous FC frame with the SC matrix. The resulting distribution of (λ) values was summarized by its percentiles for subsequent comparison. All analyses were performed using the MATLAB-based *dfcWalk* toolbox using the *TS2dFCstream*, *dFCstream2dFC* and *TS2FC* functions [6].

### Meta-Connectivity

The idea of FC can be extended to characterize the dynamic co-fluctuation across all pair-wise links in a network [6]. Accordingly, each inter-regional link is treated as a distinct unit and a correlation matrix is estimated across all N(N−1) links (for N ROIs), excluding self-connections, to capture how the fluctuations of one connection co-vary with others over time. This MC matrix provides a higher-order description of network dynamics, revealing patterns of co-fluctuation between connections in the same way that conventional FC describes correlations between nodes. More formally the MC between links *ij* and *kl* is given as:


$$\begin{array}{c} M_{ij,kl}=corr[FC_{ij}(t),FC_{kl}(t)] \end{array}$$ (3)

MC is computed using the *dFCstream2MC* function of the *dFCWalk* toolbox [6]. In order for robust statistical comparison of static FC fit and MC, we devised a bootstrap approach. The $5^{th}$, $50^{th}$ and $95^{th}$ percentiles of the sFC and MC distributions were estimated from the HCP data and model solutions for all 200 recordings. A set of 200 random integers ranging between 1 and 200 was generated (allowing for repetition). Pearson Correlation was computed between empirical and model outputs corresponding to each random draw for the sFC and MC features. This process was repeated 1,000 times to obtain a distribution of correlation coefficients for each feature.

### Neural mass modeling

Each ROI is modeled using a mean-field formulation:


$$\begin{array}{c} \frac{dS}{dt}=-\frac{S}{\tau_{s}}+(1-S_{i})\gamma R_{i}+\sigma \eta_{i}(t) \end{array}$$ (4)

$$\begin{array}{c} R_{i}=\frac{ax_{i}-b}{1-exp(-d*(ax_{i}-b))} \end{array}$$

$$\begin{array}{c} x_{i}=wJ_{N}S_{i}+J_{N}G\sum C_{ij}S_{j}+I_{0} \end{array}$$

Here, $S_{i}$ denotes the NMDA synaptic gating variable for region *i*. Total input $x_{i}$ combines 1. local recurrent coupling scaled by *w*, 2. long-range inputs weighted by the SC matrix $C_{ij}$ and globally amplified by the coupling parameter *G*, and 3. a constant external current *I*_0_. Uncorrelated noise enters through a Gaussian term $\eta_{i}(t)$ with amplitude σ and is supplied directly to the synaptic gating term. For the autonomous model (MFM) these parameters assume fixed values for each run. For the class of nonautonomous models (*tMFM*) the parameters *G*,*w*,*a*,σ were allowed to fluctuate, one at a time. These slow fluctuations were modeled as an Ornstein–Uhlenbeck (OU) process with mean (μ), mean-reversion rate (θ), and volatility (σ):


$$\begin{array}{c} dX_{t}=\theta (\mu -X_{t})\;dt+\sigma \;dW_{t}. \end{array}$$ (5)

To prevent the process from drifting into physiologically implausible low-arousal states, we imposed a lower bound equal to its mean. Specifically, after each numerical update (Euler–Maruyama), if the process fell below (μ), its value was reset to (μ):


$$\begin{array}{c} X_{t+\Delta t}=\max\;\left(X_{t+\Delta t},\;\mu \right). \end{array}$$ (6)

This “truncated” OU formulation preserves the stochastic excursions and autocorrelation structure of the OU process while enforcing a baseline level of arousal consistent with empirical observations. Each simulation was run for (950 s) with a numerical integration step of (1 ms). The initial (50 s) were discarded to eliminate transients. To reduce computational load, BOLD signals were derived by low-pass filtering the firing-rate time series below (0.2 Hz). The validity of this approximation was confirmed by comparison with a standard hemodynamic response function (S16 Fig). Finally, our simulations relied on a publicly available SC matrix derived from the HCP cohort and parcellated using the AAL atlas https://github.com/juanitacabral/NetworkModel_Toolbox.

### Model fitting

Model parameters for both the autonomous MFM and the time-varying tMFM were estimated by fitting simulated dynamics to empirical FCD. For each model, the fitting targets were the empirical FCD and FCD-speed distributions, summarized by their $5^{th}$, $25^{th}$, $50^{th}$, $75^{th}$, and $95^{th}$ percentiles, yielding a 10-dimensional feature vector capturing the full spread of temporal variability. For each BOLD recording (200 in total) the GA optimized model parameters by minimizing the Euclidean distance between this empirical vector and a corresponding vector computed from simulated data. Optimization was performed in MATLAB using the *ga* function with a population size of 50 and a maximum limit of 100 generations. Default GA operators were used, including scattered crossover and Gaussian mutation, and chromosomes within each generation were evaluated in parallel to accelerate fitness computation. A custom output function recorded the best solution and its corresponding error for each generation for each recording. For each parameter, lower and upper bounds were set based on prior parameter sweeps to constrain the solutions within plausible ranges.

## Supporting information

S1 FigError incurred by each model class: errors produced by each model class over the full dataset consisting of 200 HCP recordings.Each dot represents a single recording. Red line marks the identity line (*x* = *y*). Subtle differences in model performance are particularly visible in the tails, i.e., $\nu_{5,95}$ and $\chi_{5,95}$.(TIFF)

S2 FigHeterogeneity in model performance: tSNE based clustering on Δ AIC ($AIC_{model}-AIC_{MFM}$) identified 4 categories depicted with colors here. Bootstrap analysis of the average silhouette score indicated robust clustering.(TIF)

S3 Fig Mean AICs for 5 models from the 4 categories identified by *k*-means clustering: each cluster differed in its ability to capture data. Broadly, for 3 clusters (Category 1, 2, 3) the tMFM class was found to better account for the data to various degrees. The clustering algorithm also discovered a category of solutions where all models, including the static model, captured the on-target features equally well. This category was later found to correspond to noisy recordings. The data underlying this Figure can be found in S1 Data.
(TIF)

S4 FigTarget features for exemplars from each category identified by *k*-means clustering: exemplars were identified as datapoints with highest silhouette scores within each category.While the first 3 categories corresponded to intermediate values of Speed and FCD, the $4^{th}$ category that captured all features equally corresponded to abnormally high values of FCD and low speed indicative of noise.(TIF)

S5 Fig Meta-Connectivity for exemplars from each category identified by *k*-means clustering: exemplars were identified as datapoints with highest silhouette scores within each category. The category 4 displayed abnormally high values of Meta-Connectivity, consistent with noise. (TIF)

S6 FigModel fits for exemplars from each category: errors incurred by MFM (blue) and $tMFM_{G}$ (red) on FCD, FCD Speed, static FC and SC–FC coupling.Both MFM and $tMFM_{G}$ do equally well in explaining data, however, these recordings tend to be noisy.(TIF)

S7 Fig$5^{th}$ percentiles of Meta-Connectivity distribution for HCP data, MFM and $tMFM_{G}$ prior to global signal regression: HCP has significant negative tails, which are captured by MFM but not by $tMFM_{G}$ due to uniform comodulation of parameters in these models. This is addressed by the application of global signal regression (GSR) as shown in Figs 3–5. The data underlying this Figure can be found in S1 Data.(TIF)

S8 FigBootstrap correlation analysis: bootstrap correlation analysis across the distribution of Meta-Connectivity for the 5 model classes without global signal regression demonstrates the superiority of the $tMFM_{G}$ compared to other models. These results survive GSR correction as shown in Fig 5.(TIFF)

S9 FigEstablishing feature independence.The figure quantifies the relationship between target (fitted) and off-target (prediction) features. We find that the $tMFM_{G}$ not only captures each feature individually, but also reproduces the pattern of inter-feature correlations observed in the empirical data, substantially better than the baseline MFM. Importantly, this comparison also demonstrates that the relationship between target and off-target features is not trivial: if it were, the MFM—which is fitted only to the target features—would also recover the empirical inter-feature structure, which it clearly does not.(TIF)

S10 FigIdentifying critical transition boundaries: to delineate critical transition boundaries, a data-driven clustering procedure was employed.For every point in the parameter sweep, we computed a feature vector consisting of the $5^{th}$, $50^{th}$, and $95^{th}$ percentiles of FCD and FCD speed, along with the mean firing rate, temporal rate variability, and spatial rate variability. *K*-means clustering was then performed on these feature vectors. Boundaries in parameter space were identified using an automated procedure that detected transitions between cluster labels across the sweep. Finally, smooth exponential curves were fitted to these boundary points to define the critical transition lines shown in Fig 6.(TIFF)

S11 Fig Silhouette scores for optimal cluster identification. (TIFF)

S12 FigInferred model parameters: location of solutions superimposed on the FCD speed ($50^{th}$ percentile) phase diagram for MFM (left) and $tMFM_{G}$ (right).(TIF)

S13 FigComparative linear model.Linear models often explain time-averaged features but fail in explaining FCD fluidity. To assess how a generic linear model perfoms at capturing FCD we performed parametric exploration. Here the node dynamics is given as $\frac{dr_{j}}{dt}=-r_{j}+G\sum C_{ij}r_{j}+\sigma \eta (t)$. As is evident, the linear model offers a limited range of FCD speed before becoming unstable for G > .22.(TIFF)

S14 FigTesting for spurious correlations: we assessed the potential impact of spurious correlations in FC estimates arising from the limited sample size used for FC extraction (89 ROIs and 80 TRs per window).To this end, we first computed the FC time series using the standard sliding-window approach. For each FC matrix, we then estimated its eigenspectrum and applied a denoising step based on random matrix theory by removing eigenvalues below the Marchenko–Pastur threshold (determined by the ratio ($\frac{N_{R}OI}{winsize}$). The filtered FC matrices were subsequently reconstructed from the retained eigenmodes. Across all subjects, the correlation between FCD speed computed from the original and MP-filtered FC streams exceeded 0.9, indicating that the standard approach—despite its known limitations—captures essentially the same dynamical information, including the qualitative organization of recurrences along the FC stream.(TIFF)

S15 Fig Distribution statistics for Filtered and Unfiltered FCD estimates across 200 HCP recordings: a more detailed comparison revealed a modest effect of noise at higher percentiles of the FCD speed distribution, with faster transitions being slightly more affected than slower ones. However, this effect remains small: for example, the difference in mean values at the 95th percentile is ~0.04, and is unlikely to impact the main conclusions of the study. The data underlying this Figure can be found in S1 Data.
(TIF)

S16 FigBOLD estimation using a Hemodynamic Response Function.To convert simulated neural activity into a BOLD-like signal, we applied a standard hemodynamic response function (HRF) to the neural time series. The HRF was modeled as a canonical double-gamma function (as implemented in SPM [87]), composed of a positive gamma peak at 6 s followed by a smaller, slower undershoot at 16 s. The HRF was sampled at the native temporal resolution of the neural simulation (*dt* = 1 ms) and normalized to unit area. Neural activity was first convolved with this high-resolution HRF, ensuring that the temporal delay, dispersion, and biphasic shape of the hemodynamic response were accurately captured. After convolution, the resulting high-resolution BOLD estimate was downsampled to the fMRI sampling interval (TR = 1 s) using an anti-aliasing resampling procedure. This approach preserves the temporal fidelity of the neurovascular transformation and avoids aliasing artifacts that would occur if the neural signal were downsampled prior to HRF convolution. The figure shows parameter sweep for $\nu_{50}$ with HRF.(TIFF)

S1 Data Data underlying Figs 3 and 6, and S3, S7, and S15 Figs. (XLSX)

## Abbreviations

AD: Alzheimer’s disease
AIC: Akaike Information Criteria
DWPs: dynamic working points
FCD: functional connectivity dynamics
FC: functional connectivity
GA: Genetic Algorithm
GSR: global signal regression
HCP: Human Connectome Project
MC: Meta-Connecitivity
MFM: mean-field model
OU: Ornstein–Uhlenbeck
SC: structural connectivity
sFC: static functional connectivity
SPD: symmetric positive definite
tMFM: autonomous mean-field models.
