# Supplementary material for: Arousal-driven critical roaming reproduces human functional connectivity dynamics
Source: PLoS Biol. 2026 Sep 1;24(9):e3003916. doi: 10.1371/journal.pbio.3003916 (PMC13533355; doi:10.1371/journal.pbio.3003916)
Supplement: Supplementary file 1 [file pbio.3003916.s018.pdf]

REVIEWER #1

Thank you for inviting me to review this manuscript, in which the authors tackle a well-known gap in the modeling of resting-state functional connectivity dynamics: capturing not just "some" switching, but the full temporal complexity of empirical FCD, including its characteristic fat-tailed statistics and alternation between relatively persistent epochs and rapid reconfiguration transients. The central proposal is biologically sensible and, in my view, compelling: slow fluctuations in arousal (putatively reflecting changes in neuromodulatory tone) act as a non-autonomous drive that allows whole-brain dynamics to wander across phase boundaries near critical transition lines, thereby enriching the repertoire of accessible network states and better matching empirical FCD distributions. The work reads as solid and robust. Conceptually, it makes a clean move: rather than asking a fixed-parameter model to simultaneously produce long dwell times and heavy-tailed reconfiguration statistics, the authors let the system's effective distance to criticality drift over time via stochastic fluctuations in interpretable parameters (excitability, input gain, noise amplitude). This is exactly the kind of mechanism that feels "brain-like" in the sense that it leverages known slow state variables to shape fast network dynamics. Methodologically, the paper is strengthened by (i) identifying phase boundaries in a connectome-based model where FCD speed changes regime, (ii) formalizing arousal as stochastic modulation of key parameters (non-autonomous dynamics), and (iii) fitting to human resting-state fMRI with explicit model comparison. The headline result is also appropriately specific: arousal-driven models improve fits particularly in the fat-tailed portions of the empirical distributions, i.e., the parts that are typically hardest to match and most diagnostic of the missing mechanism.

We thank the reviewer for appreciating the importance of the central proposal, the solidity and robustness of results, the conceptual move and the pertinence of our modelling proposal centered on the role of arousal fluctuations.

From my perspective, <u>only very minor edits</u> seem necessary, and they're largely in the category of clarity and framing rather than substance

We thank the reviewer for appreciating the quality of the manuscript.

**** Clarify "arousal" operationally in the model. A short sentence making explicit that arousal is implemented purely as stochastic parameter drift (and not, e.g., an explicit brainstem node or measured physiological regressor) will help readers immediately understand the level of abstraction.***

We have now made this explicit at multiple places, indicating the simplified nature of our modeling of arousal,

ACTION: Lines added:

In the introduction: *Specifically, we simultaneously fit multiple quantitative features of human resting-state fMRI FCD using two alternative families of connectome-based whole-brain models: first, autonomous dynamical systems in which activity unfolds on a fixed landscape; and second, non-autonomous, arousal-driven models in which the dynamical landscape itself changes over time through stochastic fluctuations in model parameters, mimicking arousal-related modulation*

In the results: *To test this, we introduced an explicit arousal variable evolving as a smooth, time-dependent signal that parametrically influenced model parameters. This variable serves as a proxy for diffuse, global arousal inputs—such as those arising from brainstem and neuromodulatory systems, including the locus coeruleus—whose widespread projections influence large-scale brain dynamics.For generality, we modeled this arousal signal as a truncated Ornstein-Uhlenbeck process, defined by a baseline value, mean-reversion rate, and stochastic drive.*

**** Be explicit about which FCD summary statistics are targeted in the model comparison (e.g., distributional features, tail indices/fit range, dwell-time distributions, autocorrelation structure). Even if all are already in Methods, a one-line roadmap in the Introduction/Results will orient readers.***

We have now introduced a summary table in the Result section introducing optimization, clearly listing the used fitting target and secondary targets, with a layman definition of their meaning.

ACTION: Following table added to results

Table 1: Summary of features used for model fitting and out-of-sample validation. All distributions are summarized as the $5^{th}, 25^{th}, 50^{th}, 75^{th}$ and 95th percentiles. See methods for detailed mathematical formulae

Category	Feature	Description
Model fitting	FCD (χ)	Distribution of Functional connecitivity dynamics matrix
	FCD speed (ν)	Distribution of step lengths between subsequent, non-overlapping FC frames ($1 - corr[FC_t, FC_{t+1}]$)
Prediction	Meta-Connectivity (M)	Distribution of time-varying correlation between all edges
	SC–FC coupling (λ)	Distribution of time-varying correlation between SC and FC
	Static FC (ρ)	Correlation between empirical and simulated time-averaged FC

**** Temper the causal language around neuromodulation slightly. The phrasing "likely mediated" is already cautious; you could add one brief qualifier that this is a mechanistic hypothesis consistent with known physiology rather than directly tested here.***

We have now tried to be careful that we present our proposal as a mechanistic hypothesis that can be tested explicitly in future studies with the use of additional data such as the spatial distribution of receptors and temporal proxies such as pupil time courses etc.

ACTION: For example, we make this explicit in the discussion:

These multimodal measures provide a natural testbed for evaluating and refining the mechanistic hypotheses proposed here, and the present model—while intentionally coarse—can be readily augmented to incorporate such information in future extensions.

REVIEWER #2

The authors consider a modification to standard mean-field modeling approaches to capturing the dynamics of functional connectivity matrices in whole-brain fMRI. They contrast two categories of models, one that is autonomous and fixed (standard) and a new version [...]
This work is an important contribution to the field for the following reasons:
- The dynamics of functional connectivity are not well-understood (and are often ignored, or -- as noted in this study - categorized in ways that may be artificial)

- ***The addition of neuromodulation/drive to MFM approaches is well-motivated biologically and will open new avenues of research, several of which are discussed at the end of this study.***
- ***The critical brain hypothesis has been around for 20+ years, but remains a debate, partially because clear, functional benefits for biological function are difficult to pin down. So, criticality research is a collection of power laws and signatures that can be measured, but the impact is much harder to understand. The form of criticality put forth in this paper is a leap forward from standard models of critically tuned networks, providing a framework in which brain function (cognition, aging, degeneration) could be measured and tracked in whole-brain dynamic MFM.***

We thank the reviewer for appreciating the timeliness of the study and the qualitative novelty in our approach to relations between resting activity and the critical brain hypothesis.

Overall, this is a well-motivated and clearly executed study. It advances a novel modeling framework that takes a large step toward biological realism, while remaining conceptually interpretable.

We thank the reviewer for acknowledging the technical pertinence of our approaches.

The main weakness (concerns 1, 2) is in how features of the data are chosen for fitting to the model, which seem susceptible to noise and spurious correlation and may consequently be degrading the model fit quality. I think there are clear paths to improve this issue that should not be neglected, as this paper could be seminal in neuroimaging modeling.

We again thank the reviewer for finding our contribution valuable. We acknowledge that noise is an important factor to consider in our analysis and try to adequately address this issue in the following:

1. On spurious correlations, noise floors, and statistics of nu and metaconnectivity: My understanding is that FC speed nu is estimated as 1 - corr(FC(n), FC(n+1)), where n and n+1 index non-overlapping FC estimates over a ~60-s window. Based on 89 ROI and about ~80 TRs in a 60-s window, I would expect there to be some spurious correlation in the estimated FC, and these will be reflected in nu.

The reviewer is correct that the use of short windows can introduce spurious correlations, as discussed by Van de Ville and colleagues. This is precisely why, rather than relying on a single window size, we follow the recommendations of Battaglia et al. (2020) and Arbabyazd et al. (2020) by pooling estimates across nearby, incommensurate window sizes to construct a distribution centered around the window of interest.

In original manuscript:

To increase sampling density for relatively short recordings, speeds were computed using window lengths between 55 s and 65 s and then pooled.

This procedure enhances the contribution of consistent ("true") correlations while averaging out spurious fluctuations arising from sampling variability and aliasing. We have clarified this point in both the Methods and Discussion sections.

ACTION: Nonetheless, to further ensure that such effects do not bias our results, we performed an additional analysis based on random matrix theory filtering, as suggested by the reviewer (see response below). This confirms that residual noise contributions are minimal and do not materially affect our conclusions.

That said, based on the examples in Fig 3B, there is clear low-dimensional structure and this changes in interesting ways over time (which I understand to be the desired target of the tMFM modeling).

We interpret the reviewer's reference to "low-dimensional structure" as pointing to the recurring correlation patterns visible in the FCD matrices (e.g., original Fig. 3B). These structured recurrences are indeed the target of our modeling approach. However, we emphasize that the model is not fitted to reproduce specific instances of FCD patterns in time. Rather, it is constrained to match the statistical properties underlying these patterns, such as the distributions of FCD values and FCD speed. In this sense, the model aims to capture the generative dynamics that give rise to these low-dimensional structures, rather than reproducing their exact temporal realizations.

First, have the authors considered regularizing the FC matrices, potentially by performing SVD and dropping eigenvalues below the Marchenko-Pastur threshold?

We agree with the reviewer that a regularization procedure may in principle help reduce the contribution of spurious correlations, even if it would add an additional layer of computation, given the large number of time-resolved FC-frames. So it would be justified only whenever differences with simpler, non-regularized approaches are noticeable.

ACTION

We tested a regularized estimation procedure on a random subset of subjects; the result is shown in the figure reproduced below and now added to the manuscript as Supporting Figure (Fig. S14,15)

In short, following the suggestion of the reviewer we- 1. First extracted the dFC streams 2. Estimated the eigenspectrum for each stream, 3. Dropped eigenvalues below the Marchenko-Pastur threshold (N_roi/window_size) and finally reconstructed the FCs based on the filtered eigenvalues. Across all tested subjects, the correlation between corrected and

uncorrected estimates of FCD speed exceeded 0.9 (Fig. S14), indicating that the standard metric, although imperfect, captures essentially the same information, including the qualitative organization of recurrences along the FC stream. This is in line with previous work showing that shrinkage or regularized covariance estimation can improve robustness, especially in short-sample settings, without necessarily altering the dominant large-scale structure of the inferred connectomes (e.g. Honnorat & Habes, 2022; Xie et al., 2018). That said, upon studying the incurred error as a function of the percentiles we do notice that the higher percentiles (higher speed) tend to be affected more by the noise than the lower percentiles (Fig. S15), though this effect is very nominal.

Second, using entry-wise correlation to measure rate of change of a correlation matrix will capture the spurious correlations mentioned above. What reasons are there for using this, rather than distance measures appropriate to symmetric positive definite (SPD) matrices?

The reviewer is correct that FC(t) matrices are not generic matrices. They are symmetric correlation matrices and, aside from a limited number of weak negative entries, they are close to the positive semidefinite cone. In that sense, one could in principle adopt a more geometry-aware treatment of the FC stream, taking into account the specific structure of the space to which these matrices belong, as proposed in work on Riemannian approaches for functional connectivity and covariance-like matrices (e.g. Venkatesh et al., 2020; Simeon et al., 2022).

We nevertheless chose the simpler correlation-distance approach for two reasons. First, this is the strategy that has been used so far in the FCD literature, including by our group in the original modelling work on functional connectivity dynamics and in subsequent studies introducing and exploiting FCD speed as a marker of fluidity (Hansen et al., 2015; Battaglia et al., 2020; Lombardo et al., 2020, Hancock et. al. 2022, Alteriis et. al. 2025). Second, despite its simplicity and imperfect adaptation to the underlying matrix geometry, this metric has already shown substantial discriminative power and is the one on which the current community-level definition of FCD “speed” is effectively based. For the purposes of the present study, we therefore preferred to rely on a standard and widely accepted measure of fluidity, rather than introduce additional innovation at the level of the metric itself, given that the main novelty of our work lies in the modelling framework and in its ability to reproduce established dynamic indicators.

ACTION

Lines added in Methods:

The distance metric used above is the standard Euclidean distance, although FCD matrices are

symmetric positive definite (SPD) and therefore lie on a curved manifold. We retain the Euclidean measure for simplicity and to facilitate comparison with prior studies that adopt the same approach \cite{battaglia2020dynamic,hancock2022metastability, alteriis2025dysco}. However, we note that, in principle, alternative distance measures that respect the geometry of the SPD manifold could also be employed.

3. In Figure 3A, I am curious whether there are patterns of model performance across datasets. I would suggest making the following plot: compute \Delta AIC = AIC(tMFM_x) - AIC(MFM), generating a (# datasets) by (4 models) matrix, then cluster this matrix, either using something simple like sorting by the 'winning' model - all the tMFM_G together, all the tMFM_a together, etc. - or perhaps using PCA or hierarchical clustering. The questions are: do you see that there are groups of datasets for which specific tMFM model variants are best? Are there patterns across the tMFM models? Or are some subjects fit well by MFM, and others fit well by any tMFM? If you have multiple recordings from the same subject (and fit these separately), is best tMFM model consistent?

We thank the reviewer for this astute observation. Indeed, a cursory examination of the data did suggest to us that there exists considerable variability in how well each model class fits the data. We take this opportunity to formalize this notion. Firstly, following the reviewer suggestion we performed unsupervised clustering (k-means) on the $\Delta AICs$ calculated as (AIC_model - AIC(MFM)). This analysis yielded 4 reliable clusters (Fig. S2) (the mean silhouette score was found significant by subsequent bootstrapping analysis).

Next, we tried to understand the properties of each category as identified by unsupervised clustering. Each cluster differed in its ability to capture data. Broadly, for 3 clusters (Category 1,2,3) the tMFM class was found to better account for the data to various degrees (Fig. S3). The clustering algorithm also discovered a category of solutions where all models, including the static model, captured the on-target features equally well.

Next, we extracted the most representative subjects corresponding to each category (exemplar). These were samples with the highest silhouette score within each category. Again, while the first 3 categories corresponded to intermediate values of Speed and FCD, the 4th category that captured all features equally corresponded to abnormally high values of FCD and low speed indicative of 'junk' recordings (Fig. S4). This category also displayed abnormally high values of Metaconnectivity (Fig. S5)

In summary, this exercise revealed to us that other than the noisy category 4, all other data is better modelled using the tMFM class of models. Within the tMFM models there don't appear to be remarkable differences between the sub-variants (g,w,a,n) in terms of the AIC.

ACTION

We also add following text in the results:

To further assess heterogeneity of model performance across subjects, we performed unsupervised clustering (k-means) on relative AIC values, identifying four robust clusters. In three clusters, the tMFM class consistently outperformed the static MFM to varying degrees, whereas a fourth cluster corresponded to cases where all models performed similarly (Fig. S2,3). Examination of representative subjects revealed that this latter group was associated with atypical dynamics (high FCD χ, low FCD speed ν), suggestive of noisy or low-quality recordings (Fig. S4-6). Excluding this category, the results indicate that tMFM models provide a better account of the data, with minimal differences across their variants in terms of AIC.

4. In Figure 3C, I found the labeling to be too parsimonious, and it was difficult to interpret the spider plots. Does "Sample" mean a single recording in a single subject, or is it a sample from a recording?

We apologize for the confusion. Sample means here recording from a single subject. We now clarify it in both text and captions. Additionally we enhance clarity by splitting the original figure into 2.

ACTION:

(A) Spider plot comparing the ability of the $ MFM$ (autonomous, blue) and $tMFM_G$ (non-autonomous, red) at capturing the $5^{th},50^{th}$ and 95th percentiles of FCD (χ) and FC speed (ν) distributions for 6 sample recordings. Additionally, the spider plot indicates how the two models predict match to static FC (ρ, computed as correlation distance) and windowed SC-FC correlations (λ) and Meta-Connectivity (M) that were not used as direct fitting targets. (B). FCD recurrence matrices for the same six representative sample recordings and the corresponding best fits offered by the MFM and tMFM$_G$ models. In the bottom row, comparison between empirical and model FCD speed distributions for the same 6 recordings.

It would be helpful to show a spider plot resulting from successful model recovery (establishing what a "good fit" looks like), or at least a cartoon of 'good fit' and 'bad fit.'

We add supplementary spider plots representing the 4 classes identified earlier by clustering the relative AICs (Fig. S6)

5. In Figure 4, is there an issue with normalization in the histograms (in particular, p50, panel A?)

Indeed, we thank the reviewer for pointing it out. We now present a corrected figure with standard normalization. Each histogram corresponds to 10000 bootstrapped replicas with bin width = .01.

ACTION: Figure Updated (Figure 5 in the new draft)

6. In Figure 5, is it meaningful that the six subjects are now closer in parameter space in the dynamic MFMs, compared to the traditional MFM? The text mentions that these are representative of the full set, but it would be nice to see the density of best-fit points in the parameter space across all subjects.

We agree with the reviewer that the solutions across all subjects should be made explicit. Accordingly, we now append the following plots showing the solutions for both MFM and tMFM(G) models superimposed on the heatmaps (in the manner similar to Fig 5) to the supplementary (Fig. S12). Further, we color code the solutions in accordance with the clustering scheme as detailed in the previous response.
In addition, we further direct attention to Figure 6 (F,G,H) (originally Figure 5) where the relative distributions of solutions are summarized as box plots.

REVIEWER 3

The manuscript investigates the mechanisms causing the emergence of dynamic functional connectivity (FCD). Particularly, the manuscript studies the hypothesis that arousal can modulate functional connectivity dynamically via inter-regional coupling strength. To do so, the authors introduce a non-autonomous Wong-Wang mean-field model of whole-brain dynamics, where instead of being static, coupling strength G is allowed to fluctuate through time according to an Ornstein-Uhlenbeck random process. First, they comprehensively investigate the parameter landscape of the Wong-Wang model and characterize its bifurcations. Then, they show that non-autonomous models outperform state-of-the-art autonomous models at accounting for FC and FCD, by design, as well as at explaining static- and meta-connectivity, as an emergent property. Finally, they investigate the bifurcation landscape of the non-autonomous model and observe that critical points turn into a wider dynamical repertoire of a ‘critical roaming’ region in parameter space. This manuscript tackles a timely question following findings from Raut et al, 2025 Nature that arousal is correlated to FC dynamics. It also makes a crucial contribution to the field by endowing whole-brain models with mechanisms to

account for time fluctuations in brain dynamics beyond static FC, which are relevant clinically, for instance as pointed out in the discussion. This was a fascinating read, being clear and well-written as well as providing a comprehensive exploration of both parameter space and model-data fit.

We thank the reviewer for their appreciative remarks. We particularly thank the reviewer for pointing out the connection with the work of Raut et. al, which served as one of the motivations for this present study.

However, I have two major concerns:

1. **Likening G to arousal: while changes in G capture fluctuations in FCD, I am not convinced that it captures fluctuations that are arousal-related. Rather, changing G is a quite indirect way to modulate FCD. Changes in e.g. cholinergic modulation, as mentioned in the discussion, do not systematically or monotonically modulate regional excitability, which in turn does not systematically or monotonically modulate inter-regional connectivity strength.** ***Instead, increasing arousal reorganizes FC structure in a complex way (Raut et al, 2025 Nature)*****.**

We thank the reviewer for this important point and agree that modeling arousal as fluctuations in a single global coupling parameter (G) is a simplification. In reality, neuromodulatory systems (e.g., cholinergic and noradrenergic) act through spatially heterogeneous receptor distributions and multiple receptor subtypes, whose effects on neural excitability and synaptic transmission are diverse and often region-specific. As such, arousal-related changes are unlikely to manifest as uniform or monotonic modulations of inter-regional coupling. Our implementation of arousal as global fluctuations in (G), in line with prior work (e.g., Shine et al., *eLife*, 2018), should therefore be understood as a coarse approximation rather than a mechanistic model of neuromodulation.

That said, we would like to emphasize that even this simplified manipulation is sufficient to generate rich, non-monotonic reconfigurations of functional connectivity. In particular, the (G)–(σ) phase diagram illustrates that increasing (G) does not lead to a simple, monotonic change in FCD speed. For example, starting from a subcritical regime, increasing (G) initially stabilizes network dynamics (reducing FCD speed), but beyond a threshold drives the system into a faster, more labile regime. This results in a non-monotonic (U-shaped) relationship between coupling strength and FCD speed. Importantly, similar non-monotonic effects arise when modulating other parameters such as noise amplitude ((\sigma)), where intermediate levels can promote stability via stochastic resonance, again producing U-shaped profiles.

These results suggest that complex, non-monotonic reorganization of FC—such as those reported empirically (e.g., Raut et al., 2025)—can emerge from generic nonlinear properties of large-scale brain dynamics, even under spatially uniform parameter changes. In this sense, (G) should not be interpreted as a direct proxy for specific neuromodulatory mechanisms, but rather

as a low-dimensional control parameter capturing global shifts in the dynamical regime of the system.

Finally, the emergence of U-shaped relationships in our model is reminiscent of the classical Yerkes–Dodson law, which posits an inverted-U relationship between arousal and behavioral performance. Such nonlinear responses arise naturally from the presence of bifurcations and critical transitions in the model—features that are absent in linear systems (see Supplementary Section 1).

ACTION: We add the following lines to the discussion highlighting this aspect:

Nonetheless, neuromodulatory influences are not isolated control knobs but coordinated, low-dimensional patterns of receptor- and state-dependent action. The strong explanatory power of (G) therefore likely reflects a projection of this low-dimensional biological manifold onto model space, rather than the dominance of any single biophysical mechanism. Importantly, the nonlinear structure of the model endows it with a rich phase space, within which modulations of parameters such as G or noise amplitude (sigma) can induce transitions across regimes and give rise to phenomena such as stochastic resonance. As a result, functional connectivity dynamics exhibit non-monotonic (U-shaped) dependencies—for instance in FCD speed—reflecting an optimal intermediate regime between overly stable and overly labile dynamics. Such behavior is consistent with empirically observed reorganizations of brain activity and resonates with classical principles such as the Yerkes–Dodson law, highlighting how complex, arousal-like effects can emerge from generic nonlinear dynamics.

…….strengthening positive correlations between regions but also allowing the emergence of negative correlations (Gutierrez-Barragan et al 2022 Curr Biol).

1a. Now, it could be that the non-autonomous model accounts for this already with changing G thanks to the non-linearities in the Wong-Wang model. If so, a more detailed investigation of model-data fit to positive and negative correlations separately in relation to the value of G is warranted. For example, for negative MC values that are better fit by the non-autonomous model: is G found to be negative there? It would be interesting to see what the corresponding parameters are like for 5th (and possibly other) percentile MC.

We thank the reviewer for this insightful comment, which prompted us to perform an additional analysis.

First, we clarify that the parameter G represents inter-regional coupling and is, by construction, strictly positive, as in standard whole-brain modeling frameworks. Since G uniformly scales

positive structural connections, its transient modulation introduces a shared input across regions and therefore tends to bias correlations toward higher positive values. In this sense, fluctuations in G are inherently asymmetric: they more naturally promote increases in positive correlations than the emergence of negative ones. This limitation is visible in the Meta-Connectivity (M) distribution, particularly at the 5th percentile, where the model does not fully reproduce the strongly negative values observed in the empirical data (Fig. S7).

At the same time, changes in G are not reducible to a simple common-input effect. Excursions in G can also induce transitions between dynamical regimes, for example from slow correlated dynamics near the critical zone to faster, more decorrelated dynamics in the rate-flaring regime. We therefore asked whether removing the transient common-input component associated with homogeneous changes in G could reveal these regime-transition effects more clearly.

To test this possibility, we applied global signal regression (GSR) to the model outputs. In experimental neuroimaging, GSR is commonly used to reduce global artifacts, including motion-related or physiological components, and is also known to shift correlation distributions toward more negative values. Consistently, applying GSR to the model outputs shifted the M distribution toward more negative values, improved agreement with the empirical data, and reduced the overall absolute error.

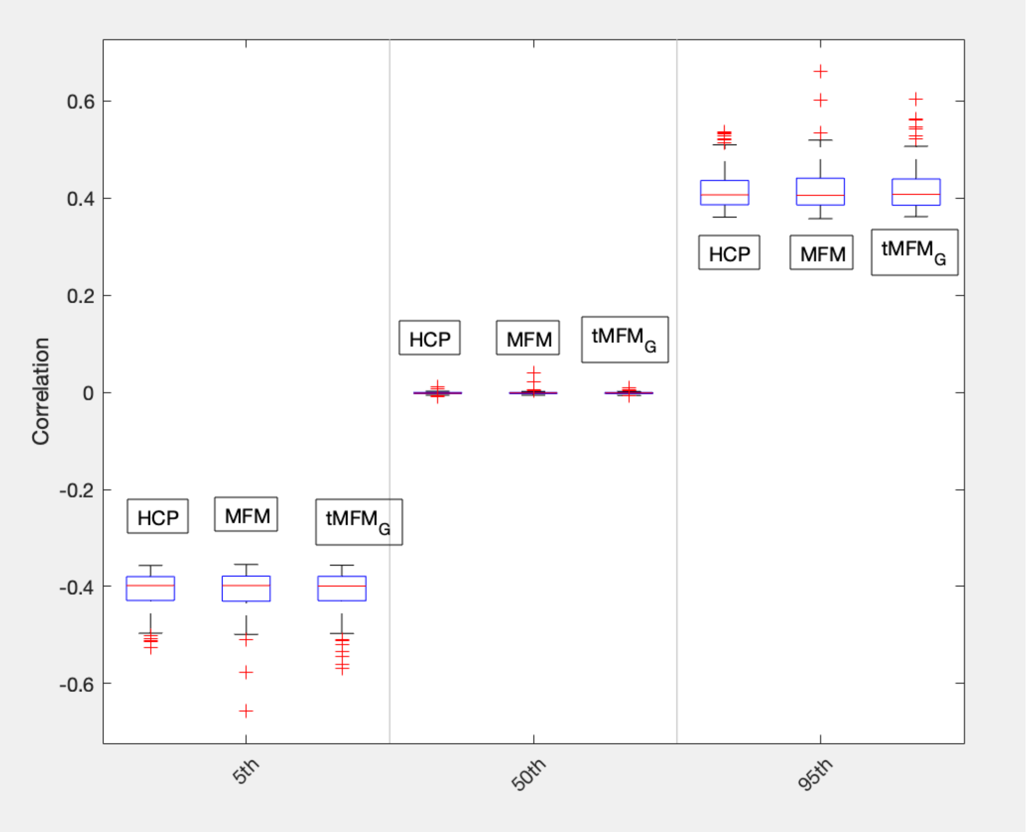

Importantly, however, bootstrap analyses (Fig 5) across subjects still showed that the tMFM(G) model provided the best overall account of Meta-Connectivity structure, particularly at the distribution tails (5th and 95th percentiles). Thus, the improved performance of tMFM(G) cannot be explained solely by a common-input effect induced by G. Rather, our interpretation is that transient increases in G both momentarily boost shared correlations and drive the system across dynamical transition boundaries, leading to qualitatively distinct network states. The superior performance of tMFM(G) therefore reflects its ability to capture these excursions across critical regime boundaries, rather than merely reproducing global correlation structure. This interpretation is further supported by the GSR analysis and is consistent with the central theoretical framework of the present study.

That said, we agree that caution is warranted when interpreting negative Meta-Connectivity correlations after GSR, since GSR is known to introduce anti-correlations. It is therefore difficult to determine whether all negative values observed after GSR reflect genuine underlying dynamics or are partly induced by preprocessing. Crucially, however, even without GSR, the autonomous MFM already exhibits negative Meta-Connectivity correlations (Fig. S8). This shows that anti-correlations can arise intrinsically from the model dynamics, particularly near phase-transition boundaries where the system operates in a highly nonlinear regime.

A detailed investigation of the precise origin of these anti-correlations would require a dedicated study and lies beyond the scope of the present work. We nevertheless thank the reviewer for raising this important point, which allowed us to clarify this aspect of the model's behavior. We also thank the reviewer for suggesting the relevant citation by Gutiérrez-Barragán et al. (2022, Current Biology), which we have now incorporated into the manuscript.

ACTION: *We now correct Figure 5 (originally figure 4) to include GSR corrected Meta-Connectivity, while also retaining the original figure in the supplementary (Fig. S8). Moreover, we add lines in the results and discussion section.*

In Results:

We note that, in the preceding analysis, both the sFC and Meta-Connectivity were extracted after performing a global signal regression (GSR). This choice is motivated by the fact that in the tMFM, a neuromodulation of model parameters is implemented as a uniform scaling of the parameter across all the nodes, which has the tendency of biasing positive correlations due to common inputs. Applying GSR mitigates this common-input effect, enabling a more balanced comparison of correlation structure. Importantly, however, even in the absence of GSR—where absolute errors are larger—the bootstrap correlation analysis (which is insensitive to absolute scaling) continues to demonstrate the superiority of the tMFM_{G} (see Supplementary). This indicates that the improved performance of tMFM_{G} is not solely driven by global correlation biases, but reflects its ability to capture meaningful structure in the data.

In the Discussion:

First, we turn to the role of negative correlations (anti-correlations) in FCD, which are increasingly recognized as functionally meaningful \cite{gutierrez2022unique}. A limitation of our framework is that neuromodulatory effects are modeled as uniform modulations of the model parameters, which biases the system toward increased positive correlations due to shared fluctuations across regions. Applying global signal regression (GSR) partially mitigates this effect and introduces negative values in the tail of the meta-connectivity distribution (Fig. 3,4); however, these anti-correlations cannot be unambiguously attributed to intrinsic dynamics, as GSR itself is known to induce such effects \cite{murphy2009impact}. Notably, the static MFM already exhibits negative meta-connectivity tails even in the absence of GSR (see Supplementary), suggesting that at least part of this phenomenon may arise from the underlying nonlinear dynamics. Disentangling these contributions represents an important direction for future work.

1b. To claim to model cholinergic modulation impacts on whole-brain dynamics, the manuscript could take inspiration from models introduced for that exact purpose in the literature: Cakan et al, 2023 Cogn Comput; Goldman et al, 2023 Front Comput Neurosci. Would varying spike-frequency adaptation strength through time, instead of varying G, yield similar effects?

We thank the reviewer for pointing us to these relevant studies and have incorporated them where appropriate in the revised manuscript. We agree that models explicitly incorporating neuromodulatory mechanisms—such as those in Cakan et al. 2023 Cogn Comput and Goldman et al. 2023 Frontiers in Computational Neuroscience—provide a more mechanistic account of how cholinergic modulation may influence large-scale dynamics.

In its current form, the mean-field model (MFM) used here does not include an explicit spike-frequency adaptation mechanism. Instead, neuromodulatory effects are captured in a coarse-grained manner via fluctuations in the global coupling parameter (G). As the reviewer suggests, modulating adaptation strength over time would constitute a more biologically grounded alternative, given that neuromodulators such as acetylcholine are known to regulate adaptation currents and can induce state transitions (e.g., between persistent and UP–DOWN dynamics, as observed during different vigilance states).

We expect that time-varying adaptation could, in principle, produce qualitatively similar effects on functional connectivity dynamics (FCD), insofar as both mechanisms act to shift the system between dynamical regimes. However, the relationship is unlikely to be trivial or one-to-one, as adaptation primarily alters local excitability and temporal integration properties, whereas (G) directly modulates inter-regional coupling. Establishing the extent to which these two control mechanisms yield comparable FCD signatures would require a systematic exploration of parameter space.

We have therefore added to the Discussion that an important extension of the present framework would be to replace the current MFM with a mean-field reduction of models that explicitly incorporate adaptation, such as the Adaptive Exponential Integrate-and-Fire model. Such an approach would allow for a more mechanistic investigation of neuromodulatory effects on whole-brain dynamics, including their impact on FCD. We view this as an important direction for future work.

ACTION : Following lines added to the discussion section

Similarly, node dynamics could be extended to include additional targets of neuromodulatory action. In particular, mean-field models incorporating spike-frequency adaptation—known to be modulated by acetylcholine—provide a biologically grounded mechanism for shaping large-scale dynamics, including the emergence of Up–Down state transitions characteristic of NREM sleep. Such extensions could be implemented using mean-field reductions of models like the Adaptive Exponential Integrate-and-Fire model, enabling a more mechanistic investigation of how neuromodulation influences functional connectivity dynamics

2. The manuscript uses static FC and meta-connectivity as metrics that the model isn't fitted on to demonstrate that the model captures other aspects of the data than those it's designed to capture. But how correlated are static FC and meta-connectivity to the metrics the model is designed to fit? There should be a verification that this correlation isn't so strong that the model's ability to account for these metrics is essentially trivial.

Fig. S9 quantifies the relationship between target (fitted) and off-target (unfitted) features. We find that the tMFM(G) not only captures each feature individually, but also reproduces the pattern of inter-feature correlations observed in the empirical data, substantially better than the baseline MFM. Importantly, this comparison also demonstrates that the relationship between target and off-target features is not trivial: if it were, the MFM—which is fitted only to the target features—would also recover the empirical inter-feature structure, which it clearly does not.

More generally, the metrics considered here (FCD, FCD speed, and meta-connectivity) are not mathematically coupled in any simple way. For example, random temporal scrambling of FC frames preserves meta-connectivity but destroys the temporal organization captured by FCD and FCD speed. This illustrates that these features probe distinct aspects of the data. Consistent with this, prior work (e.g., Arbabyazd et al. Network Neuroscience, 2023) has shown the existence of "genuine trimers" in meta-connectivity—higher-order relationships that cannot be reduced to pairwise FC structure alone.

To further quantify this independence, we computed the mutual information between target and off-target features. This analysis shows that only ~6% of the variance in off-target features (including MC and SC–FC distributions) can be explained by the fitted features (FCD and FCD speed). This result further indicates that the model's ability to reproduce off-target features is not

a trivial consequence of fitting the target metrics, but reflects its capacity to capture additional, independent structure in the data.

ACTION Following text added to results

Further, we assessed whether the model's ability to reproduce off-target features arises trivially from fitting the target metrics by analyzing their statistical relationships. The $tMFM_{G}$ captures both individual features and their inter-feature correlation structure significantly better than the baseline MFM, indicating that these relationships are non-trivial (Fig. S9).

<u>….Is there a strawman model that could account for FCD and FC speed as well as the non-autonomous model but not for static FC or meta-connectivity?</u>

We draw the attention of the reviewer to Figure 5 (originally Fig. 4) which precisely addresses this point. As is clearly seen, time-dependent models differ substantially in their ability to capture aspects of MC and static FC even though all of them do equally well at capturing the on-target features (FCD and FC Speed) as evidenced by the AIC analysis presented in Fig 3A. This further supports the claim that the improvements could not have solely arisen due to the correlations between features, but rather arise from the underlying dynamics.

Minor points:
I didn't understand in what sense the model was non-autonomous.

In dynamical systems theory, a standard distinction is made between autonomous and non-autonomous systems. An autonomous system is one whose evolution is governed solely by its current state, with no explicit dependence on time, typically written as ($\dot{x}$ = f(x)). Such systems are invariant under time translation, meaning their qualitative behavior does not depend on the absolute time at which they are observed. In contrast, a non-autonomous system has dynamics that depend explicitly on time, ($\dot{x}$ = f(x,t)), thereby breaking time-translation invariance. Equivalently, this can be viewed as a system in which the underlying vector field evolves over time due to external forcing or time-dependent parameters.

In contrast to conventional autonomous formulations with fixed coupling parameters, we model, as example, the coupling (G) as a latent stochastic process following Ornstein–Uhlenbeck dynamics. Importantly, G(t) evolves independently of the neural state, without feedback from the brain dynamics. This induces a stochastically time-varying modulation of the effective connectivity, rendering the observed system non-autonomous when viewed in the neural state space, while remaining autonomous in the joint ((x, G)) augmented system.

FC speed, meta-connectivity: Given how important this is to the manuscript, at least an overview of how they're computed in the results text or main figures is warranted.

We introduce an additional table (Table 1 in results; see reviewer 1 also) to further clarify the definition of the derived features.

Fig. 3D: the text is very small and hard to read without zooming in substantially and looking at only a small subset of plots at a time.

We now split Fig 3 into two to make it more readable.
