# Supplementary material for: Arousal-driven critical roaming reproduces human functional connectivity dynamics
Source: PLoS Biol. 2026 Sep 1;24(9):e3003916. doi: 10.1371/journal.pbio.3003916 (PMC13533355; doi:10.1371/journal.pbio.3003916)
Supplement: Supplementary file 2 [file pbio.3003916.s019.pdf]

RESPONSE TO REVIEWERS

Reviewer #2: The reviewers have adequately addressed the points in my critique, confirming that their results are robust to the regularization of FC matrices as well as exploring possible sub-types across recordings in terms of MFM vs tMFM[x] fit performance.

I recommend publication.

We thank the reviewer for their insightful comments that inspired important analysis.

Reviewer #3 [Trang-Anh Nghiem]: Reviewer comments have been thoroughly addressed, and the manuscript is close to ready for publication.

We thank the reviewer for the critical comments and helping us improve the article.

My only minor concern is the usage of the term 'arousal' throughout the manuscript, such as in sub-section titles in the Results, to describe the mechanism modulated in simulations. As explained in my review from the previous round, there is not a one-to-one correspondence between arousal and global coupling G, as neuromodulators driving arousal also modulates local excitability at the regional level (as mentioned in the manuscript) and spike-frequency adaptation and its associated timescales. <u>I would prefer if when referring strictly to the model, the manuscript used wording like 'dynamical global coupling' or even 'arousal via dynamical coupling' rather than just 'arousal', which is misleading. It is fine to say that results are consistent with arousal-related fluctuations seen empirically, and that G fluctuations are reminiscent of arousal fluctuations, but the wording needs to be a little more careful.</u>

We agree that the use of the term *arousal* requires careful qualification. In previous revisions, we sought to emphasize that the relationship between neuromodulatory systems and the model parameters is necessarily complex, and that our implementation represents a simplified approximation of these processes. For instance, the following lines in the earlier draft highlight this subtlety

<u>Nonetheless, neuromodulatory influences are not isolated control knobs but coordinated, low-dimensional patterns of receptor- and state-dependent action. The strong explanatory power of (G) therefore likely reflects a projection of this low-dimensional biological manifold onto model space, rather than the dominance of any single biophysical mechanism.</u>

While this abstraction cannot capture all known effects of arousal-related neuromodulation, it nevertheless provides useful insight into the organization of functional connectivity, particularly its temporal dynamics. That said, we agree that some instances in the manuscript may suggest a stronger correspondence between arousal and the model parameter than is warranted,

including the example highlighted by the reviewer. To address this concern, we have renamed the principal Results section from *"Arousal as a global modulatory drive in the mean-field model"* to *"<u>Arousal-like</u> global modulatory drive in the mean-field model."* We believe this change more accurately conveys that fluctuations in global coupling should be interpreted as a simplified, arousal-related mechanism rather than as a direct representation of arousal itself, and it establishes this distinction from the outset of the discussion.

Similarly we add the following note to further underscore this nuance:

<u>We emphasize that this formulation is intended as a simplified modeling abstraction of arousal-related neuromodulatory influences, capturing their aggregate, slow effects on model parameters rather than providing a direct or one-to-one representation of physiological arousal. In what follows, we continue to refer to this mechanism as "arousal" for simplicity</u>

An even more minor detail related to this: in the sentence "As a result, functional connectivity dynamics exhibit non-monotonic (U-shaped) dependencies—for instance in FCD speed" it would be helpful to explicitly refer to the figure(s) and/or reference(s) showing this. <u>I believe this is in Figure 2F, for large values of a, but given that this non-monotonic relation is not seen in panels E, G, or H, it may be better to change the wording to "can exhibit non-monotonic [...]" unless I am missing other evidence, in which case those should be explicitly cited.</u>

The reviewer is correct. We change the wording accordingly and cite the relevant figure.

That said, dynamical global coupling as a mechanism is interesting in itself, and I appreciate that the manuscript now shows a rigorous and comprehensive investigation of how this mechanism can shape brain dynamics.

We thank the reviewer again for appreciating this point.
