# Supplementary material for: Arousal-driven critical roaming reproduces human functional connectivity dynamics
Source: PLoS Biol. 2026 Sep 1;24(9):e3003916. doi: 10.1371/journal.pbio.3003916 (PMC13533355; doi:10.1371/journal.pbio.3003916)
Supplement: Peer review history

# Editor report: Decision Letter 0

**DOI:** 10.1371/journal.pbio.3003916.r001

**Authors:** Taylor Hart, PhD

30 Jan 2026

Dear Dr Pathak,

Thank you for submitting your manuscript entitled "The critical roaming hypothesis: arousal-driven transitions across critical lines reproduce human functional connectivity dynamics" for consideration as a Research Article by PLOS Biology.

Your manuscript has now been evaluated by the PLOS Biology editorial staff, as well as by an academic editor with relevant expertise, and I am writing to let you know that we would like to send your submission out for external peer review.

However, before we can send your manuscript to reviewers, we need you to complete your submission by providing the metadata that is required for full assessment. To this end, please login to Editorial Manager where you will find the paper in the 'Submissions Needing Revisions' folder on your homepage. Please click 'Revise Submission' from the Action Links and complete all additional questions in the submission questionnaire.

Once your full submission is complete, your paper will undergo a series of checks in preparation for peer review. After your manuscript has passed the checks it will be sent out for review. To provide the metadata for your submission, please Login to Editorial Manager (https://www.editorialmanager.com/pbiology) within two working days, i.e. by Feb 01 2026 11:59PM.

If your manuscript has been previously peer-reviewed at another journal, PLOS Biology is willing to work with those reviews in order to avoid re-starting the process. Submission of the previous reviews is entirely optional and our ability to use them effectively will depend on the willingness of the previous journal to confirm the content of the reports and share the reviewer identities. Please note that we reserve the right to invite additional reviewers if we consider that additional/independent reviewers are needed, although we aim to avoid this as far as possible. In our experience, working with previous reviews does save time.

If you would like us to consider previous reviewer reports, please edit your cover letter to let us know and include the name of the journal where the work was previously considered and the manuscript ID it was given. In addition, please upload a response to the reviews as a 'Prior Peer Review' file type, which should include the reports in full and a point-by-point reply detailing how you have or plan to address the reviewers' concerns.

During the process of completing your manuscript submission, you will be invited to opt-in to posting your pre-review manuscript as a bioRxiv preprint. Visit http://journals.plos.org/plosbiology/s/preprints for full details. If you consent to posting your current manuscript as a preprint, please upload a single Preprint PDF.

Feel free to email us at plosbiology@plos.org if you have any queries relating to your submission.

Kind regards,

Taylor

Taylor Hart, PhD,

Associate Editor

PLOS Biology

thart@plos.org

# Peer review: Decision Letter 1

**DOI:** 10.1371/journal.pbio.3003916.r002

**Authors:** Taylor Hart, PhD

3 Apr 2026

Dear Dr Pathak,

Thank you for your patience while your manuscript "The critical roaming hypothesis: arousal-driven transitions across critical lines reproduce human functional connectivity dynamics" was peer-reviewed at PLOS Biology. It has now been evaluated by the PLOS Biology editors, an Academic Editor with relevant expertise, and by several independent reviewers. We apologize again for the delay in reaching a decision, which was related to the need to recruit an additional reviewer after one reviewer dropped out.

In light of the reviews, which you will find at the end of this email, we would like to invite you to revise the work to thoroughly address the reviewers' reports.

As you will see, both reviewers found the study to be conceptually compelling and generally well-executed. However, Reviewer 2 raised several technical points and questioned the justifications for some of the methodological choices, while Reviewer 1 commented on areas requiring clarification or softening of statements. In your revision, you should thoroughly address the reviewers' points.

Given the extent of revision needed, we cannot make a decision about publication until we have seen the revised manuscript and your response to the reviewers' comments. Your revised manuscript is likely to be sent for further evaluation by all or a subset of the reviewers.

In addition to these revisions, you will need to complete some formatting changes, which you will receive in a follow up email. A member of our team will be in touch with a set of requests shortly.

We expect to receive your revised manuscript within 3 months. Please email us (plosbiology@plos.org) if you have any questions or concerns, or would like to request an extension.

At this stage, your manuscript remains formally under active consideration at our journal; please notify us by email if you do not intend to submit a revision so that we may withdraw it.

**IMPORTANT - SUBMITTING YOUR REVISION**

Your revisions should address the specific points made by each reviewer. Please submit the following files along with your revised manuscript:

1. A 'Response to Reviewers' file - this should detail your responses to the editorial requests, present a point-by-point response to all of the reviewers' comments, and indicate the changes made to the manuscript.

*NOTE: In your point-by-point response to the reviewers, please provide the full context of each review. Do not selectively quote paragraphs or sentences to reply to. The entire set of reviewer comments should be present in full and each specific point should be responded to individually, point by point.

You should also cite any additional relevant literature that has been published since the original submission and mention any additional citations in your response.

2. In addition to a clean copy of the manuscript, please also upload a 'track-changes' version of your manuscript that specifies the edits made. This should be uploaded as a "Revised Article with Changes Highlighted" file type.

*Re-submission Checklist*

When you are ready to resubmit your revised manuscript, please refer to this re-submission checklist: https://plos.io/Biology_Checklist

To submit a revised version of your manuscript, please go to https://www.editorialmanager.com/pbiology/ and log in as an Author. Click the link labelled 'Submissions Needing Revision' where you will find your submission record.

Please make sure to read the following important policies and guidelines while preparing your revision:

*Published Peer Review*

Please note while forming your response, if your article is accepted, you may have the opportunity to make the peer review history publicly available. The record will include editor decision letters (with reviews) and your responses to reviewer comments. If eligible, we will contact you to opt in or out. Please see here for more details:

https://blogs.plos.org/plos/2019/05/plos-journals-now-open-for-published-peer-review/

*PLOS Data Policy*

Please note that as a condition of publication PLOS' data policy (http://journals.plos.org/plosbiology/s/data-availability) requires that you make available all data used to draw the conclusions arrived at in your manuscript. If you have not already done so, you must include any data used in your manuscript either in appropriate repositories, within the body of the manuscript, or as supporting information (N.B. this includes any numerical values that were used to generate graphs, histograms etc.). For an example see here: http://www.plosbiology.org/article/info%3Adoi%2F10.1371%2Fjournal.pbio.1001908#s5

*Blot and Gel Data Policy*

We require the original, uncropped and minimally adjusted images supporting all blot and gel results reported in an article's figures or Supporting Information files. We will require these files before a manuscript can be accepted so please prepare them now, if you have not already uploaded them. Please carefully read our guidelines for how to prepare and upload this data: https://journals.plos.org/plosbiology/s/figures#loc-blot-and-gel-reporting-requirements

*Protocols deposition*

To enhance the reproducibility of your results, we recommend that if applicable you deposit your laboratory protocols in protocols.io, where a protocol can be assigned its own identifier (DOI) such that it can be cited independently in the future. Additionally, PLOS ONE offers an option for publishing peer-reviewed Lab Protocol articles, which describe protocols hosted on protocols.io. Read more information on sharing protocols at https://plos.org/protocols?utm_medium=editorial-email&utm_source=authorletters&utm_campaign=protocols

Thank you again for your submission to our journal. We hope that our editorial process has been constructive thus far, and we welcome your feedback at any time. Please don't hesitate to contact us if you have any questions or comments.

Sincerely,

Taylor

Taylor Hart, PhD,

Associate Editor

PLOS Biology

thart@plos.org

------------------------------------

REVIEWS:

Reviewer #1: Thank you for inviting me to review this manuscript, in which the authors tackle a well-known gap in the modeling of resting-state functional connectivity dynamics: capturing not just "some" switching, but the full temporal complexity of empirical FCD, including its characteristic fat-tailed statistics and alternation between relatively persistent epochs and rapid reconfiguration transients. The central proposal is biologically sensible and, in my view, compelling: slow fluctuations in arousal (putatively reflecting changes in neuromodulatory tone) act as a non-autonomous drive that allows whole-brain dynamics to wander across phase boundaries near critical transition lines, thereby enriching the repertoire of accessible network states and better matching empirical FCD distributions.

The work reads as solid and robust. Conceptually, it makes a clean move: rather than asking a fixed-parameter model to simultaneously produce long dwell times and heavy-tailed reconfiguration statistics, the authors let the system's effective distance to criticality drift over time via stochastic fluctuations in interpretable parameters (excitability, input gain, noise amplitude). This is exactly the kind of mechanism that feels "brain-like" in the sense that it leverages known slow state variables to shape fast network dynamics. Methodologically, the paper is strengthened by (i) identifying phase boundaries in a connectome-based model where FCD speed changes regime, (ii) formalizing arousal as stochastic modulation of key parameters (non-autonomous dynamics), and (iii) fitting to human resting-state fMRI with explicit model comparison. The headline result is also appropriately specific: arousal-driven models improve fits particularly in the fat-tailed portions of the empirical distributions, i.e., the parts that are typically hardest to match and most diagnostic of the missing mechanism.

From my perspective, only very minor edits seem necessary, and they're largely in the category of clarity and framing rather than substance:

* Clarify "arousal" operationally in the model. A short sentence making explicit that arousal is implemented purely as stochastic parameter drift (and not, e.g., an explicit brainstem node or measured physiological regressor) will help readers immediately understand the level of abstraction.

* Be explicit about which FCD summary statistics are targeted in the model comparison (e.g., distributional features, tail indices/fit range, dwell-time distributions, autocorrelation structure). Even if all are already in Methods, a one-line roadmap in the Introduction/Results will orient readers.

* Temper the causal language around neuromodulation slightly. The phrasing "likely mediated" is already cautious; you could add one brief qualifier that this is a mechanistic hypothesis consistent with known physiology rather than directly tested here.

Overall: the paper's contribution is clear, the mechanism is plausible, and the modeling results seem to land exactly where they should — improving the tail behavior that standard near-critical autonomous models tend to miss. If the full manuscript maintains the same level of rigor as this abstract suggests, I would be comfortable recommending acceptance pending only minor editorial adjustments.

Reviewer #2: The authors consider a modification to standard mean-field modeling approaches to capturing the dynamics of functional connectivity matrices in whole-brain fMRI. They contrast two categories of models, one that is autonomous and fixed (standard) and a new version, in which modulatory drive reshapes the dynamics (non-autonomous). These models are fit to FC matrices estimated over 1-minute windows in human fMRI recordings; a key aspect that they aim to explain is how FC matrices change over time. Standard (autonomous) mean-field models fail to capture intermittent 'jumps' in FC structure, while the driven MFM is flexible enough to capture these dynamics. The implication of this finding is that whole-brain dynamics can be steered by neuromodulators, and rather than hovering near a 'critical point' the dynamics move around an "ignition line."

This work is an important contribution to the field for the following reasons:

-The dynamics of functional connectivity are not well-understood (and are often ignored, or -- as noted in this study - categorized in ways that may be artificial)

-The addition of neuromodulation/drive to MFM approaches is well-motivated biologically and will open new avenues of research, several of which are discussed at the end of this study.

-The critical brain hypothesis has been around for 20+ years, but remains a debate, partially because clear, functional benefits for biological function are difficult to pin down. So, criticality research is a collection of power laws and signatures that can be measured, but the impact is much harder to understand. The form of criticality put forth in this paper is a leap forward from standard models of critically tuned networks, providing a framework in which brain function (cognition, aging, degeneration) could be measured and tracked in whole-brain dynamic MFM.

Comments:

Overall, this is a well-motivated and clearly executed study. It advances a novel modeling framework that takes a large step toward biological realism, while remaining conceptually interpretable. The main weakness (concerns 1, 2) is in how features of the data are chosen for fitting to the model, which seem susceptible to noise and spurious correlation and may consequently be degrading the model fit quality. I think there are clear paths to improve this issue that should not be neglected, as this paper could be seminal in neuroimaging modeling.

1.On spurious correlations, noise floors, and statistics of nu and metaconnectivity: My understanding is that FC speed nu is estimated as 1 - corr(FC(n), FC(n+1)), where n and n+1 index non-overlapping FC estimates over a ~60-s window. Based on 89 ROI and about ~80 TRs in a 60-s window, I would expect there to be some spurious correlation in the estimated FC, and these will be reflected in nu. That said, based on the examples in Fig 3B, there is clear low-dimensional structure and this changes in interesting ways over time (which I understand to be the desired target of the tMFM modeling). First, have the authors considered regularizing the FC matrices, potentially by performing SVD and dropping eigenvalues below the Marchenko-Pastur threshold? Second, using entry-wise correlation to measure rate of change of a correlation matrix will capture the spurious correlations mentioned above. What reasons are there for using this, rather than distance measures appropriate to symmetric positive definite (SPD) matrices?

2.Related to 1, it seems the noise floor for meta-connectivity would also be quite high, and that replicable structure in the MC matrix would arise from interactions between low-dimensional structure in FC matrices. I suspect that regularization and clearly motivated distance measures would be appropriate here as well, rather than entry-wise comparisons.

3.In Figure 3A, I am curious whether there are patterns of model performance across datasets. I would suggest making the following plot: compute \Delta AIC = AIC(tMFM_x) - AIC(MFM), generating a (# datasets) by (4 models) matrix, then cluster this matrix, either using something simple like sorting by the 'winning' model - all the tMFM_G together, all the tMFM_a together, etc. - or perhaps using PCA or hierarchical clustering. The questions are: do you see that there are groups of datasets for which specific tMFM model variants are best? Are there patterns across the tMFM models? Or are some subjects fit well by MFM, and others fit well by any tMFM? If you have multiple recordings from the same subject (and fit these separately), is best tMFM model consistent?

Minor comments/questions

4.In Figure 3C, I found the labeling to be too parsimonious, and it was difficult to interpret the spider plots. Does "Sample" mean a single recording in a single subject, or is it a sample from a recording? It would be helpful to show a spider plot resulting from successful model recovery (establishing what a "good fit" looks like), or at least a cartoon of 'good fit' and 'bad fit.'

5.In Figure 4, is there an issue with normalization in the histograms (in particular, p50, panel A?)

6.In Figure 5, is it meaningful that the six subjects are now closer in parameter space in the dynamic MFMs, compared to the traditional MFM? The text mentions that these are representative of the full set, but it would be nice to see the density of best-fit points in the parameter space across all subjects.

# Author comment: Author response to Decision Letter 2

**DOI:** 10.1371/journal.pbio.3003916.r003

7 May 2026

Attachment Submitted filename: Answers to reviewers.pdf

# Peer review: Decision Letter 2

**DOI:** 10.1371/journal.pbio.3003916.r004

**Authors:** Taylor Hart, PhD

11 Jun 2026

Dear Dr Pathak,

Thank you for your patience while we considered your revised manuscript "The critical roaming hypothesis: arousal-driven transitions across critical lines reproduce human functional connectivity dynamics" for publication as a Research Article at PLOS Biology. This revised version of your manuscript has been evaluated by the PLOS Biology editors, the Academic Editor, and two of the original reviewers.

Based on the reviews, we are likely to accept this manuscript for publication, provided you satisfactorily address the remaining points raised by Reviewer 3. Please also make sure to address the following data and other policy-related requests.

IMPORTANT: Please ensure that your next revision addresses the following points:

--------------------------

**Title:

We would like to modify your paper's title to conform with our stylistic guidelines (which normally do not allow for punctuation). Is either of the following alternative formulations acceptable to you?

1. Arousal-driven transitions across critical lines reproduce human functional connectivity dynamics

2. Stochastic arousal fluctuations modulate critical phase boundaries to shape brain network dynamics

**Financial disclosure statement:

Please confirm that all relevant grant numbers have been included in the Financial Disclosure Statement in the manuscript details. Please also add links to the funding agencies in the statement.

**Data Statement:

As you the original neuroimaging data were not generated for this paper, please do not mention the HCP data in the Data Statement on the submission form (but please do still include this information in the manuscript text).

Please also update the data statement to mention where the new data underlying the figures can be found (see below).

**Data and Code provision:

We require that you provide the numerical data underlying some of the figures. For the following figure panels, please provide the relevant data either in the online supplement, or as a new supplementary excel file "S1 Data" (filename: S1_Data.xlsx). You can put data from different panels/figures in different tabs of the same overall file. Please also include a note in the relevant figure legends on where these data can be found, e.g. “The data underlying this Figure can be found in S1 Data” or “The data underlying this Figure can be found in https://doi.org/10.5281/zenodo.XXXXX”

3ABC

6DEFGH

S3

S7

S15

Thank you for providing the underlying code in GitHub. However, because Github depositions can be readily changed or deleted, please make a permanent DOI’d copy (e.g. in Zenodo) and provide this URL in the manuscript and Data Availability Statement. Please also make sure that you choose a license for code reuse.

**Supplement format:

Please upload the supplementary items individually as "Supporting Information" files.

-------------------------

As you address these items, please take this last chance to review your reference list to ensure that it is complete and correct. If you have cited papers that have been retracted, please include the rationale for doing so in the manuscript text, or remove these references and replace them with relevant current references. Any changes to the reference list should be mentioned in the cover letter that accompanies your revised manuscript.

In addition to these revisions, you may need to complete some formatting changes, which you will receive in a follow up email. A member of our team will be in touch with a set of requests shortly. If you do not receive a separate email within a few days, please assume that checks have been completed, and no additional changes are required.

We expect to receive your revised manuscript within two weeks.

To submit your revision, please go to https://www.editorialmanager.com/pbiology/ and log in as an Author. Click the link labelled 'Submissions Needing Revision' to find your submission record. Your revised submission must include the following:

- a cover letter that should detail your responses to any editorial requests, if applicable, and whether changes have been made to the reference list

- a Response to Reviewers file that provides a detailed response to the reviewers' comments (if applicable, if not applicable please do not delete your existing 'Response to Reviewers' file.)

- a track-changes file indicating any changes that you have made to the manuscript.

NOTE: If Supporting Information files are included with your article, note that these are not copyedited and will be published as they are submitted. Please ensure that these files are legible and of high quality (at least 300 dpi) in an easily accessible file format. For this reason, please be aware that any references listed in an SI file will not be indexed. For more information, see our Supporting Information guidelines:

https://journals.plos.org/plosbiology/s/supporting-information

*Published Peer Review History*

Please note that you may have the opportunity to make the peer review history publicly available. The record will include editor decision letters (with reviews) and your responses to reviewer comments. If eligible, we will contact you to opt in or out. Please see here for more details:

https://plos.org/published-peer-review-history/

*Press*

Should you, your institution's press office or the journal office choose to press release your paper, please ensure you have opted out of Early Article Posting on the submission form. We ask that you notify us as soon as possible if you or your institution is planning to press release the article.

*Protocols deposition*

To enhance the reproducibility of your results, we recommend that if applicable you deposit your laboratory protocols in protocols.io, where a protocol can be assigned its own identifier (DOI) such that it can be cited independently in the future. Additionally, PLOS ONE offers an option for publishing peer-reviewed Lab Protocol articles, which describe protocols hosted on protocols.io. Read more information on sharing protocols at https://plos.org/protocols?utm_medium=editorial-email&utm_source=authorletters&utm_campaign=protocols

Please do not hesitate to contact me should you have any questions.

Sincerely,

Taylor

Taylor Hart, PhD,

Associate Editor

thart@plos.org

PLOS Biology

REVIEWS

Reviewer #2: The reviewers have adequately addressed the points in my critique, confirming that their results are robust to the regularization of FC matrices as well as exploring possible sub-types across recordings in terms of MFM vs tMFM[x] fit performance.

I recommend publication.

Reviewer #3 [Trang-Anh Nghiem]: Reviewer comments have been thoroughly addressed, and the manuscript is close to ready for publication. My only minor concern is the usage of the term 'arousal' throughout the manuscript, such as in sub-section titles in the Results, to describe the mechanism modulated in simulations. As explained in my review from the previous round, there is not a one-to-one correspondence between arousal and global coupling G, as neuromodulators driving arousal also modulates local excitability at the regional level (as mentioned in the manuscript) and spike-frequency adaptation and its associated timescales. I would prefer if when referring strictly to the model, the manuscript used wording like 'dynamical global coupling' or even 'arousal via dynamical coupling' rather than just 'arousal', which is misleading. It is fine to say that results are consistent with arousal-related fluctuations seen empirically, and that G fluctuations are reminiscent of arousal fluctuations, but the wording needs to be a little more careful.

An even more minor detail related to this: in the sentence "As a result, functional connectivity dynamics exhibit non-monotonic (U-shaped) dependencies—for instance in FCD speed" it would be helpful to explicitly refer to the figure(s) and/or reference(s) showing this. I believe this is in Figure 2F, for large values of a, but given that this non-monotonic relation is not seen in panels E, G, or H, it may be better to change the wording to "can exhibit non-monotonic [...]" unless I am missing other evidence, in which case those should be explicitly cited.

That said, dynamical global coupling as a mechanism is interesting in itself, and I appreciate that the manuscript now shows a rigorous and comprehensive investigation of how this mechanism can shape brain dynamics.

# Author comment: Author response to Decision Letter 3

**DOI:** 10.1371/journal.pbio.3003916.r005

26 Jun 2026

Attachment Submitted filename: Response to reviewers-1.pdf

# Editor report: Decision Letter 3

**DOI:** 10.1371/journal.pbio.3003916.r006

**Authors:** Taylor Hart, PhD

8 Jul 2026

Dear Dr Pathak,

Thank you for the submission of your revised Research Article "Arousal-driven critical roaming reproduces human functional connectivity dynamics" for publication in PLOS Biology. On behalf of my colleagues and the Academic Editor, Choong-Wan Woo, I am pleased to say that we can in principle accept your manuscript for publication, provided you address any remaining formatting and reporting issues. These will be detailed in an email you should receive within 2-3 business days from our colleagues in the journal operations team; no action is required from you until then. Please note that we will not be able to formally accept your manuscript and schedule it for publication until you have completed any requested changes.

Please take a minute to log into Editorial Manager at http://www.editorialmanager.com/pbiology/, click the "Update My Information" link at the top of the page, and update your user information to ensure an efficient production process.

PRESS

We frequently collaborate with press offices. If your institution or institutions have a press office, please notify them about your upcoming paper at this point, to enable them to help maximise its impact. If the press office is planning to promote your findings, we would be grateful if they could coordinate with biologypress@plos.org. If you have previously opted in to the early version process, we ask that you notify us immediately of any press plans so that we may opt out on your behalf.

We also ask that you take this opportunity to read our Embargo Policy regarding the discussion, promotion and media coverage of work that is yet to be published by PLOS. As your manuscript is not yet published, it is bound by the conditions of our Embargo Policy. Please be aware that this policy is in place both to ensure that any press coverage of your article is fully substantiated and to provide a direct link between such coverage and the published work. For full details of our Embargo Policy, please visit http://www.plos.org/about/media-inquiries/embargo-policy/.

Thank you again for choosing PLOS Biology for publication and supporting Open Access publishing. We look forward to publishing your study.

Sincerely,

Taylor

Taylor Hart, PhD,

Associate Editor

PLOS Biology

thart@plos.org
